# Intraspecific variability of the saccular and utricular otoliths of the hatchetfish *Argyropelecus hemigymnus* (Cocco, 1829) from the Strait of Messina (Central Mediterranean Sea)

**DOI:** 10.1371/journal.pone.0281621

**Published:** 2023-02-14

**Authors:** Claudio D’Iglio, Sergio Famulari, Marco Albano, Alex Carnevale, Dario Di Fresco, Mariachiara Costanzo, Giovanni Lanteri, Nunziacarla Spanò, Serena Savoca, Gioele Capillo

**Affiliations:** 1 Department of Chemical, Biological, Pharmaceutical and Environmental Sciences, University of Messina, Messina, Italy; 2 Department of Biomedical, Dental and Morphological and Functional Imaging, University of Messina, Messina, Italy; 3 Department of Veterinary Sciences, University of Messina, Messina, Italy; University of Split, Faculty of science, CROATIA

## Abstract

Mesopelagic species are enjoining increasing attention due to the growing impact of fisheries activities on deep marine biocenosis. Improving the knowledge base on mesopelagic species is required to enhance their conservation due to the knowledge gaps regarding many species and families. In this context, otoliths can be fundamental to assessing their life history, ecomorphological adaptation to the deep environment and stock composition. The present paper aims to explore the saccular and utricular otoliths morphology and intra-specific variability of the hatchetfish, *Argyropelecus hemigymnus*, from the Strait of Messina. *Lapilli* and *sagittae* were collected from 70 specimens and separated into four size classes. Morphometric, shape and SEM investigations were performed to describe their morphology, contours, and external structural organization, also studying their intraspecific variability related to sample sizes and differences between otolith pairs. Results showed an otolith morphology different from those reported in the literature with fluctuating asymmetry in *sagittae* and *lapilli* belonging to Class IV, and a high otolith variability between all the size classes. Data herein described confirm the otoliths singularity of the population from the Strait of Messina, shaped by a unique marine environment for oceanographic and ecological features.

## Introduction

The vertebrates’ inner ear represents a highly specialized organ for sound detection, motion/position measuring, and equilibrium regulation [1, 2]. All the vertebrates (except for the jawless) share a similar inner ear morphology, with one ear for side, each characterized by three semicircular canals. In most non-mammalian vertebrates, these canals present three otolithic end organs (*utricle*, *saccule*, *lagena*). Within each of these are calcium carbonate crystals that in teleost fishes solidify in single acellular masses, called otoliths (respectively *lapillus*, *sagitta*, *asteriscus*) [3]. These are characterized by continued growing during the entire fish’s lifetime, with a daily deposition of new material [4, 5]. Their isolation from the external environment and their capability to be metabolically inert make them an essential tool for fish life history studies [4, 6]. Especially *sagittae* (the largest among otoliths in non-otophysan species [7]) have been extensively used in many research fields (e.g., fisheries science [8–12], ecology [13–20], taxonomy [21–25], palaeontology [26–28] and eco-geochemistry [29]) due to their species-specific morphology [25, 30, 31], their persistence in ancient sediments [32] and stomach contents of ichthyophages predators [13–16], and their inter-specific variability in morphology, microstructure and microchemical composition [33, 34]. Despite *lapilli* and *asterisci* being widely described in many species [35–37], there is relatively less information, if compared with *sagittae*, regarding their morphology and diversity, especially in marine teleost [38]. *Lapilli* has been broadly used for the identification of otophysan fishes (being larger than *sagittae*) [39, 40], but due to their generally small size, their low persistence in geological layers and predators’ stomach contents, and their almost completely unknown intra and interspecific diversity, the knowledge base on these otoliths is still limited, especially regarding Mediterranean bony fishes.

The Mediterranean Sea is a semi-enclosed basin characterized by enhanced biodiversity and a high anthropogenic impact related to pollution, fisheries activities, and urbanization. The growing impact of human activities (especially fisheries [41–44]) on Mediterranean deep environments has led the scientific community to focus on meso- and bathypelagic communities. Due to their vertical migrations and trophic relationships, these play a fundamental ecological role in the energy flowing and carbon transport between different marine domains [45–54]. Mesopelagic fishes show a great abundance in biomass, being the main component of the deep scattering layer (DSL) and mesopelagic zone, and the most abundant vertebrates on earth for their density and diffusion in all the Oceans [55, 56]. Several studies have been focused worldwide on these species’ distribution, biology, biodiversity and ecology [51, 56–59], also investigating morphology, microstructures and growth of *sagittae* [57, 60–65]. Despite this, the knowledge base on mesopelagic fishes remains scarce, with several gaps regarding the biology and eco morphology of many species and families. Due to their deep distribution, these teleosts are mainly sampled with expensive methods, such as trawling (being large specimens abundant in trawling discards and by-catch) or other nets for micronekton sampling (e.g., Isaacs-Kidd Midwater Trawl Net, Environmental Sensing System, young fish trawl) [58, 66]. However, the small dimensions of these fishes (often smaller than large trawl mashes), added to their high mobility and patchy distribution (which often is related to specimens’ ontogenetic stage, time of the day and season), increase the difficulties in obtaining representative fresh samples useful to investigate their life histories, biodiversity, and population dynamics without bias.

In this context, the Strait of Messina takes on great importance. It is characterized by an intense hydrodynamism, with very strong upwelling currents strictly related to tidal phases [67–69]. These moon-related phenomena, combined with the strong winds blowing in the area and the daily vertical movements performed by mesopelagic micronekton, cause a natural stranding, sometimes even massive, of deep fauna along the Sicilian and Calabrian coasts [70, 71]. These peculiar events were well documented and studied from the end of the 800’ century, making the Strait of Messina one of the main Mediterranean geographical areas to study and investigate the mesopelagic fauna [71–81].

The current paper aims to examinae the morphology, morphometry, shape, and external textural organization of saccular (*sagittae*) and utricular (*lapilli*) otoliths of the half-naked hatchetfish, *Argyropelecus hemigymnus*, Cocco, 1829, from the Strait of Messina, also investigating the occurrence of bilateral asymmetry and their intraspecific variability related to specimens’ total length and weight. The family Sternoptychidae (hatchetfishes) belongs to the order Stomiiformes and represents one of the most abundant teleost’s family in biomass and abundance of the mesopelagic zone worldwide [82–84]. It includes 73 valid species distributed in all the Oceans, characterized by bodies usually smaller than 100 mm (total length, TL), with several photophores species specifically distributed on their surface, and great intergeneric morphological variability [85]. In the Mediterranean Sea, this family includes three genera (*Argyropelecus*, *Maurolicus* and *Valenciennellus*), with the *Argyropelecus* genus (deep-bodied hatchetfishes) composed of three species (*A*. *hemigymnus*, *Argyropelecus olfersii*, Cuvier, 1829, and *Argyropelecus aculeatus*, Valenciennes, 1850) [86]. *A*. *hemigymnus* is distributed worldwide and, like the other deep-bodied hatchetfishes species, inhabits deep marine environments (up to 1000 m of depth), forming shoals and aggregations [87]. They generally stay in the deep during the day to avoid predation (being preyed on by a large number of predators belonging to several taxa [47, 79, 88–91]), performing vertical migrations for trophic porpoises at night, following their preys (mainly euphausiids for larger specimens, and copepods for smaller specimens) [47, 92].

According to previous studies performed in the Strait of Messina [70, 71], *A*. *hemigymnus* is among the most numerically relevant species for abundance and frequency of stranding during the entire year. This large number of available specimens is useful to obtain information on the eco-morphological adaptations and life history of this species due to the high inter-regional variability of life histories in mesopelagic teleost [59, 93]. Therefore, obtaining new data on hathcetfishes’ saccular and utricular otoliths’ morphology and intra-specific variability, applying techniques never applied before on this species, such as SEM and shape analysis, is essential to improve the knowledge base on this rare and still poorly studied mesopelagic teleost, exploring ecomorphological adaptation to deep marine habitats. This represents a fundamental step to fully understanding the teleosts’ inner ear functioning and how its morphology change in relation to the environmental and fishes’ life cycle. The data obtained in the present study increase the information on the inner ear of a cosmopolitan and ecologically essential species, investigating its intra-specific variability, which can be an expression of environmental biophysical effects or an indicator for environmental stress, nutritional condition and water column seasonal variations (such as Fluctuating asymmetry) [61, 94], also adding new data on the poorly understood, but not the less important for being so, utricular otoliths [95, 96]. Moreover, all this information can pave the way to further comparisons with other populations of the same species from different geographical areas, clarifying the effects of environmental (such as currents and physiochemical water features) and ecological conditions on mesopelagic fishes’ otoliths.

## Materials and methods

### Sampling area

A total of 70 individuals of *A*. *hemigymnus* were sampled before the sunshine (to avoid the action of scavenger predators, such as bees, rats, cats, and seagulls) on the Sicilian coast of the Strait of Messina in March 2022. Specimens were stranded on the shore due to the high hydrodynamism and strong winds acting in the area.

**Fig 1:**
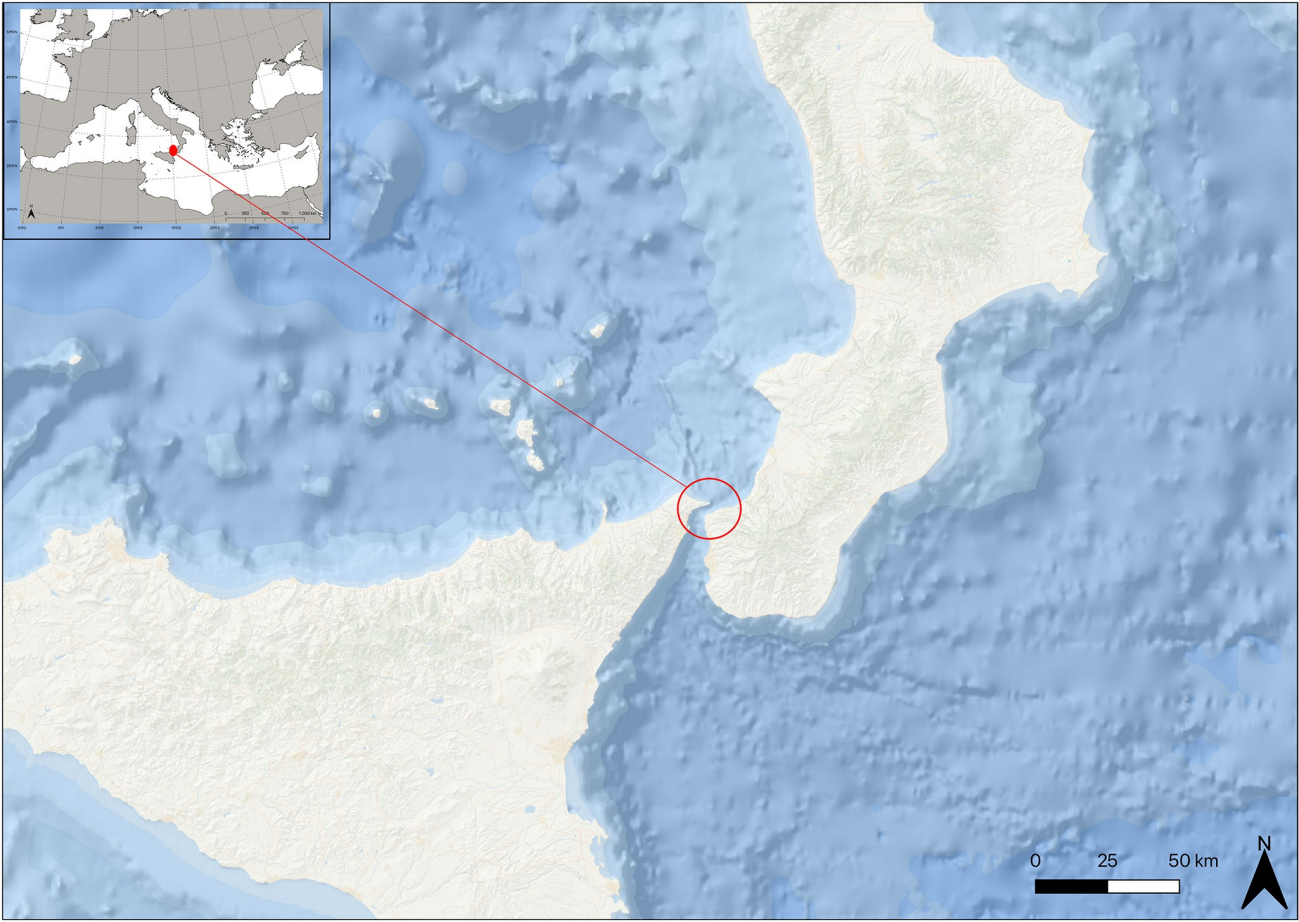
Maps of the studied area with the Strait of Messina reported in the red circle and the Mediterranean Sea in the insert. Data source: QGIS Development Team. QGIS Geographic Information System (version 3.26). https://qgis.org/en/site/.

Indeed, the Strait of Messina (central Mediterranean Sea) is located at the junction between Tyrrhenian and Ionian Seas, separating the Italian peninsula from Sicily (Fig 1). This peculiar position makes it a meeting and colliding area between two water masses with different physic-chemical properties [68, 69]. The narrow passage of the Strait (only around 3 km separates the Italian and Sicilian coast at the nearest point) enlarges toward the Tyrrhenian Sea at the north and the Ionian Sea at the south, with an enhanced morpho-bathymetrical irregularity of the bottom. A shallower central area (the saddle, -70/-90 m of depth) divides the Strait into two deeper zones: one toward the Tyrrhenian Sea (the northern Strait’s exit, -100/-400 m of depth) and one toward the Ionian Sea (the southern Strait’s exit, until -1000 m of depth). The narrowest and shallowest Strait zone amplifies the water’s volume from the two basins, producing strong currents acting in the area (with velocity until 3 m s^−1^) [97, 98]. The water masses get mixed, flowing one on the other, with dynamics regulated by tidal phases, according to their physic-chemical properties. The semi-diurnal currents inversions cause a difference in elevation between the Ionian and Tyrrhenians Seas (when one is in high tide, the other is in low tide, and vice versa). This gradient drives a large volume of water to pass across the Strait’s saddle from South to North and vice versa, in alternating phases with opposite directions, every 5–6 hours.

This intense hydrodynamism results from the upwelling of deep water from the Ionian Sea, which is one of the main causes of marine organisms’ stranding. The intense flowing of waters from the deep, added to the Straits physiography, allows a quick transport of deep fauna toward the surface. This rapid bathymetric change causes shock or even death in the organisms that, moved by wave, wind and current, strain on the shore [62, 70, 71]. This natural phenomenon is also influenced by seasonality, wind direction, moon phases and different ecological and biological features of the species, which regulate their movement along the water column. Indeed, the vertical daily migrations of several mesopelagic species toward the shallower marine strata enhance the risk of being swept by the current and transported too quickly on the surface.

### Samples processing, images elaboration and morphometric analysis

Once sampled, all the specimens were transferred still fresh in the laboratory, where each one was measured (total length, TL) and weighed (total weight, TW). The individuals were assigned to four size classes, according to their TL. Class I comprised all those with a TL between 10 and 20 mm, Class II between 20 and 30 mm, Class III between 30 and 40 mm and Class IV greater than 40 mm. Each left and right sagittal and utricular otolith was sampled and polished from tissue remains using 3% H_2_O_2_ for 15 minutes and Milli-Q water. Once dried, they were photographed twice (one photo for each otolith face) under a stereomicroscope Zeiss Discovery V8 equipped with Axiocam 208 colour camera (Carl Zeiss, Jena, Germany), being later stored in Eppendorf microtubes. One *sagitta* and one *lapillus* for each size class were chosen for SEM analysis.

ImageJ 1.48p software [99] was used to perform several measurements on otoliths images and convert them into binary format for contour extraction. For each *sagitta* and *lapillus*, it was measured the maximum otolith length (OL, mm), the maximum otolith width (OW, mm), the otolith perimeter (OP, mm) and the otolith surface (OS, mm^2^). It was also calculated the ratio of otolith length to the total fish length (OL/TL) to investigate how otoliths increase in length in relation to fish total length. In order to evaluate how the shape of *sagittae* and *lapilli* varied in the different size classes, several shapes indices were calculated for each otolith according to the literature [57, 100–104]: circularity (C = OP^2^/OS), rectangularity (Re = OS/[OL×OW]), ellipticity (E = (OL–OW)/(OL+OW)), aspect ratio (AR = OW/OL%), form factor (FF = 4πOS/OP^2^) and roundness (Ro = 4OS/πOL^2^). Circularity and roundness show how the otolith’s shape resembles a perfect circle, considering minimum values 1 and 4π, respectively. Rectangularity gives information on how otolith length and width vary in relation to the surface, with the value of 1 assumed by a perfect square. Ellipticity indicates if changes in the otolith’s axis are proportional, giving information on how it is similar to an ellipse, resulting in 0 for a perfect circle. Aspect ratio, the ratio between width and length, gives information on how the otolith is elongated; the larger aspect ratio value, the more elongated the otolith. The form factor indicates how the otolith’s contour is similar to a circle, with values ranging between 0 and 1, where 1 indicates a perfect circle.

### Shape analysis

Shape analysis based on the outlines of the collected otoliths was performed using shape R, an open-access package that runs on R software (RStudio 2022.07.1 Build 554; R Gui 4.1.3 2022.03.10). This R package was specially designed to study otolith shape variation among bony fishes populations or species [105]. Each taken picture of *sagittae* and *lapilli* was first binarized using ImageJ software (version 1.53k freely available at https://imagej.nih.gov/ij/) and subsequently classified based on fish size class and otolith side. The outlines were detected through a specific function of shape R, with the grayscale threshold value set at 0.05 (intensity threshold). The contours thus extracted were linked to a data file containing information about the specimens analyzed (e.g., fish length and body weight). Otolith measurements (i.e., length, width, perimeter, and area) for each size class were calculated using the getMeasurements function based on outlines previously detected. Wavelet and Fourier coefficients were extracted and adjusted through proper functions of the shape R package to define the allometric relationships between otolith shapes and fish lengths.

### SEM analysis

A total of four *sagittae* and four *lapilli* (one *sagitta* and one *lapillus* for size classes) were investigated through SEM analysis with a Zeiss EVO MA10 at the acceleration voltage of 20Kv. Firstly 70% alcohol for 48 hours was used to fix the samples. After this, they were soaked in a series of alcohol (from 70% to 100%, one hour for each passage) to dehydrate them. One stub (SEM-PT-F-12) covered by conductive adhesive tables (G3347) was used to place otoliths, avoiding the critical drying point. They were left at 28° for 12h, and finally, before the observation at SEM, a layer of 20 nm gold palladium was deposited to sputter coated them.

### Data analysis

Univariate and multivariate statistical analyses were conducted using Prism V.8.2.1 (Graph-pad Software Ltd., La Jolla, CA 92037, USA), R vegan package V.2.5, and PAST V. 2.756.

Morphological parameters were analyzed using an unpaired t-test to highlight any significant differences between the right and left sides of the otoliths.

Differences in morphological parameters between specimens of different ontogenetic classes were analyzed using a one-way analysis of variance (one-way ANOVA). The correlation between the measured parameters and fish weight and total length was also tested using the Pearson correlation coefficient.

Additionally, wavelet coefficients were used to analyze shape variation among the left and right sides of *lapilli* and *sagittae* and between ontogenetic classes using an ANOVA-like permutation test, to determine differences in otolith contours. Moreover, shape coefficients were subjected to a Linear Discriminant Analysis (LDA) to obtain an overview of the differences in otolith shape between the size classes examined. All analyses were conducted on *lapilli* and *sagittae*. The significance level was set at P < 0.05.

## Results

### Morphometric and shape analysis

#### Sagittae

According to the terminology used by Tuset, Nolf and Assis [21, 25, 38], *A*. *hemigymnus* specimens showed tall *sagittae*, higher than wider, characterized by an oval to angled shape and a vertical axis longer than the horizontal one. The dorsal region was tapered, with an asymmetrical shape and a rounded extremity. The ventral region was globular with a symmetrical shape. Dorsal and ventral rims were smooth and convex. The external face was smooth and convex-shaped, while the internal face was also smooth but concave. *Rostrum* and *antirostrum* were inconspicuous, very short and round. In Table 1 there were reported the morphometric mean values obtained for *sagittae*, divided into investigated size classes.

**Table 1:** Morphometric mean values of *sagittae*, standard deviation (s.d.) and minimums (Min.) and maximus (Max.) values divided for the size classes investigated: Maximum otolith length (OL, mm), the maximum otolith width (OW, mm), otolith perimeter (OP, mm) and otolith surface (OS, mm^2^), the ratio of otolith length to the total fish length (OL/TL), circularity (C = OP^2^/OS), rectangularity (Re = OS/[OL×OW]), ellipticity (E = OL–OW/OL+OW), aspect ratio (AR = OW/OL%), form factor (FF = 4πOS/OP^2^) and roundness (Ro = 4OS/πOL^2^).

	CLASS I			CLASS II			CLASS III			CLASS IV		
	Mean	s.d.	Min.—Max.	Mean	s.d.	Min.—Max.	Mean	s.d.	Min.—Max.	Mean	s.d.	Min.—Max.
**OL**	0.309	0.029	0.237–0.347	0.394	0.035	0.333–0.476	0.718	0.056	0.631–0.872	0.846	0.044	0.754–0.923
**OW**	0.379	0.032	0.316–0.434	0.518	0.051	0.424–0.600	0.519	0.043	0.437–0.610	0.570	0.032	0.531–0.636
**OP**	1.260	0.117	0.989–1.474	1.755	0.190	1.408–2.129	2.167	0.154	1.946–2.497	2.573	0.160	2.289–2.917
**OS**	0.091	0.015	0.059–0.116	0.161	0.028	0.122–0.215	0.284	0.039	0.234–0.385	0.374	0.037	0.310–0.444
**OL / TL**	0.022	0.004	0.015–0.030	0.017	0.002	0.012–0.022	0.021	0.002	0.017–0.028	0.020	0.001	0.018–0.022
**C**	17.597	1.235	16.227–20.486	19.229	2.091	16.048–23.779	16.591	0.547	15.661–18.089	17.731	0.938	15.482–19.889
**Re**	0.771	0.015	0.745–0.798	0.786	0.015	0.764–0.814	0.760	0.018	0.723–0.794	0.772	0.021	0.735–0.816
**E**	-0.541	0.091	-0.782–-0.432	-0.400	0.097	-0.579–-0.167	0.513	0.103	0.344–0.785	0.742	0.078	0.580–0.872
**AR**	1.229	0.051	1.157–1.336	1.312	0.071	1.161–1.497	0.724	0.047	0.614–0.795	0.675	0.039	0.621–0.748
**FF**	0.718	0.048	0.614–0.775	0.661	0.071	0.529–0.784	0.759	0.024	0.695–0.803	0.711	0.037	0.632–0.812
**Ro**	0.798	0.028	0.750–0.833	0.763	0.038	0.678–0.881	1.340	0.107	1.160–1.599	1.460	0.095	1.272–1.598

**Fig 2:**
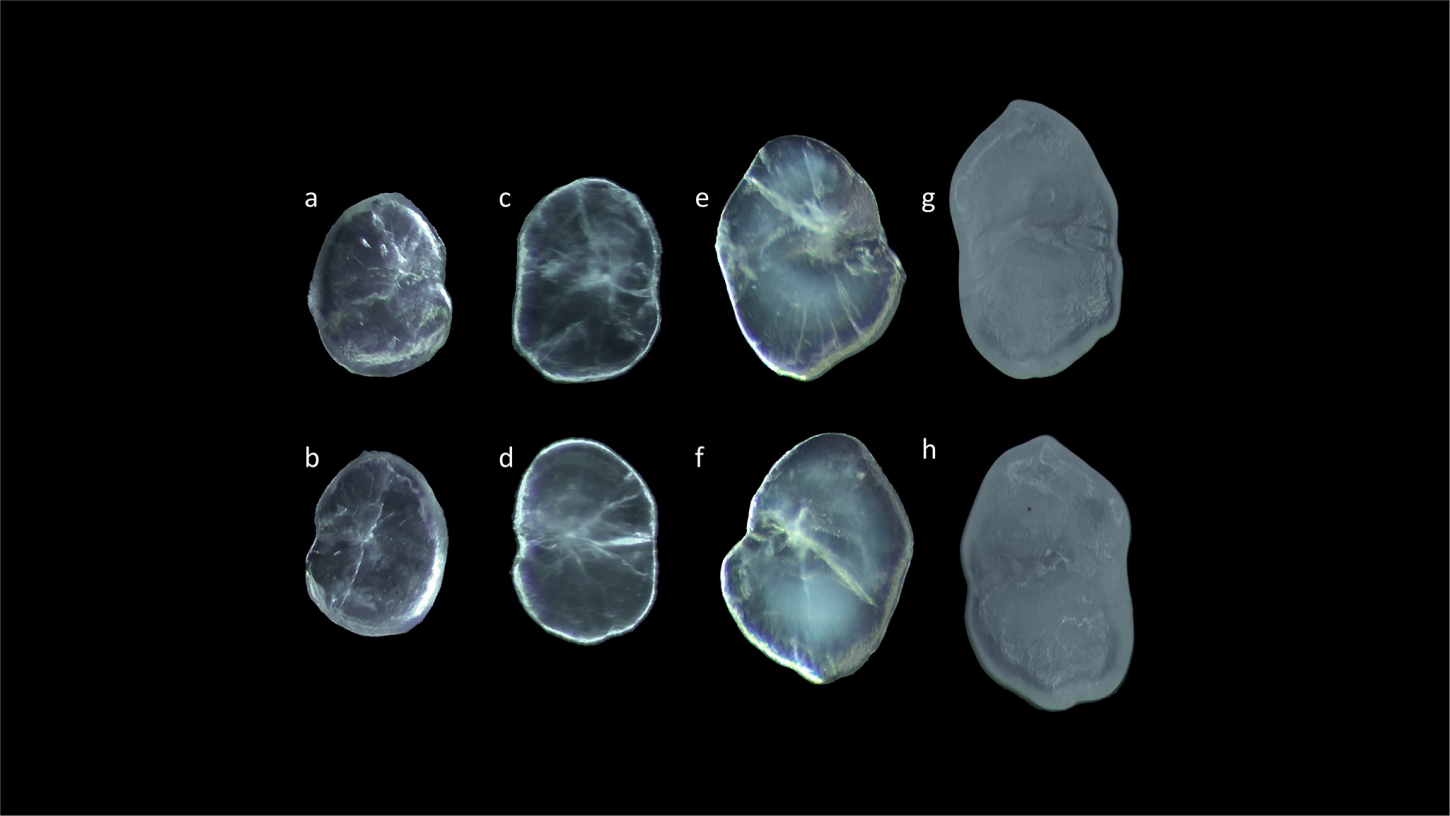
Stereoscope images of left (a,c,e,g) and right (b,d,,f,h) *sagittae* inner surfaces belonging to size Classes I (a,b), II (c,d), III (e,f) and IV (g,h).

Concerning morphological differences between size classes (Fig 2a–2h), *sagittae* of specimens belonging to Class I showed a marked asymmetry between dorsal and ventral regions, with a very enhanced globular shape, especially in the ventral one, and a marked *excisura ostii*, which became gradually most inconspicuous in the other size Classes. The dorsal region became increasingly tapered in Classes II, III and IV, with a slightly triangular shape in Classes III and IV, characterized by crenate margins and a most increased otoliths’ width than length. In Class IV, the ventral region became less globular, with an angled shape characterized by an irregular angular rim and a most enhanced symmetry with the dorsal region. The traits that have remained constant among the size classes were *rostrum* and *antirostrum* (short and round in all the size classes) and the heterosulcoid *sulcus acusticus*.

ANOVA showed significant differences for almost all the morphometric measurements of the *sagittae* between the four size classes examined (p < 0.05) (S1 Table). A significant correlation between the body weight and total length of the specimens and the morphometries of the *sagittae* was observed for all parameters except for A/(OLxOH) (P > 0.05) (S2 and S3 Tables). The morphometrical parameters did not show significant differences between the right and left *sagittae* for each size class investigated (p > 0.05).

**Fig 3:**
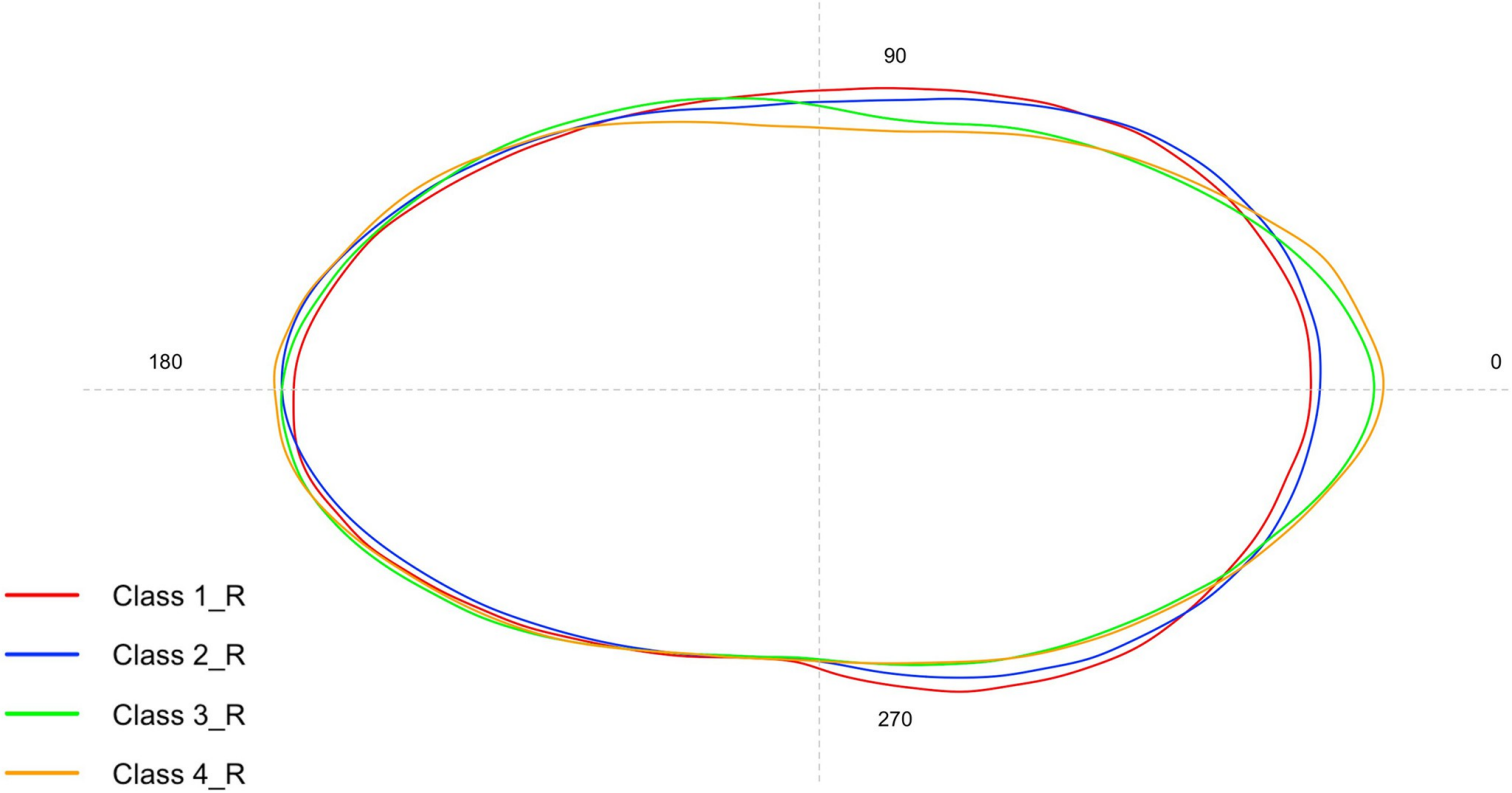
Mean shape of right *sagittae* contours belonging to the four investigated size classes.

The graph in Fig 3 represents the mean otolith shape comparison among different size classes for right *sagittae* obtained through standardized Wavelet coefficients. The quality of both Wavelet and Fourier reconstruction was estimated by comparing the deviations from the otolith outlines, with the value 15 sets as the maximum number of Fourier harmonics to be shown (S1 Fig). The mean and standard deviation of calculated coefficients was plotted using the gplots R package to assess how the variation of Wavelet coefficients depends on the position along the outline (S2 Fig).

**Fig 4:**
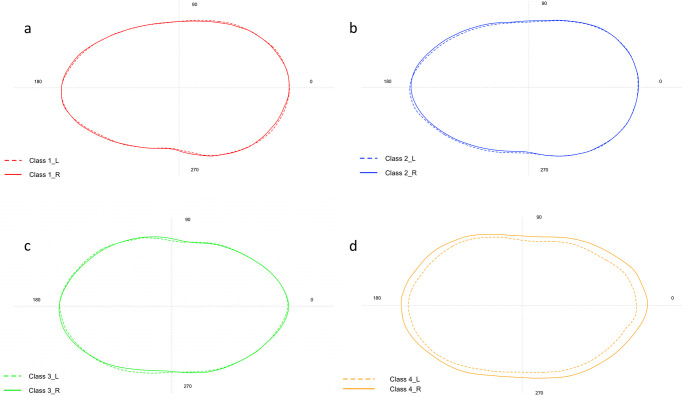
Comparison between the mean shape of left and right *sagittae* contours belonging to Class I (a), Class II (b), Class III (c) and Class (IV).

**Fig 5:**
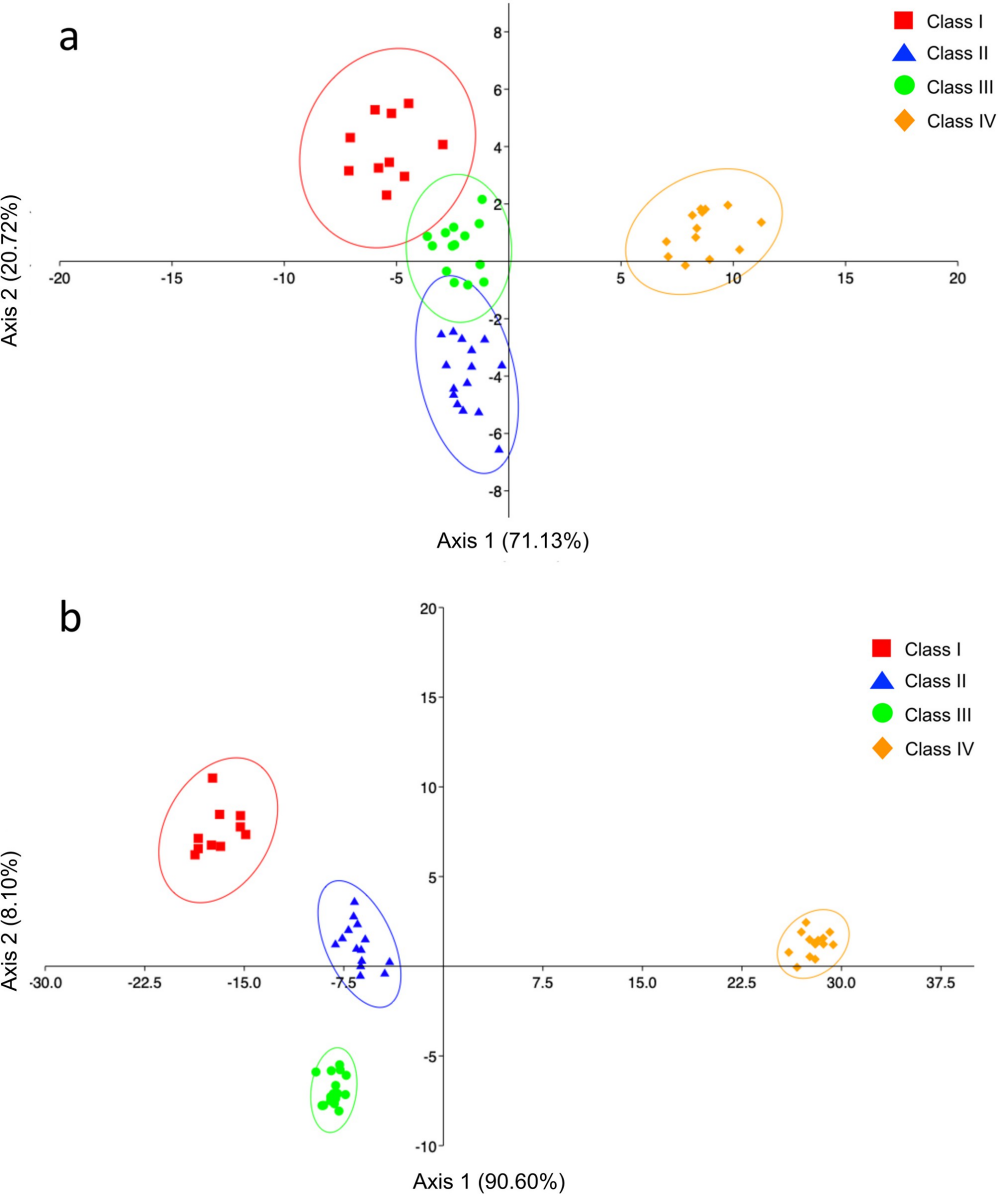
Linear Discriminant Analysis (LDA) computed between the size classes analyzed, calculated on Wavelet Fourier descriptors obtained by the left side (a) and right (b) of *sagittae*. Ellipses includes 95% confidence interval.

The shape analysis showed a significant difference between the right and left side of the *sagittae* for all sizes classes (S4 Table) except for Class II (p = 0.18) (Fig 4a–4d). Furthermore, significant variability of the boundaries was observed between size classes for both the right and left sides (p = 0.001). LDA highlighted how the contours of the class IV sagittas are markedly separated from the left and right contours obtained for the other size classes, as shown in Fig 5 (Axis 1 71.13% and 90.6%, respectively).

#### Lapilli

According to the terminology used by Assis [35, 38], the *lapilli* of *A*. *hemigymnus* showed a non-clupeiform type morphology, with a globular anterior region and a slender posterior region. The internal and external margins were smooth, convex shaped and asymmetrical, with curved rims with different degrees of bending. The *Extremum posterior* was tapered with a triangular shape and oriented horizontally, while the *extremum anterior* was rounded. *Prominentia marginalis* was large and rounded, and *gibbus maculae* was slender and small. *Sulcus lapilli* was superficial and very thin. Table 2 reported the morphometric mean values obtained for *lapilli*, divided into the investigated size classes.

**Table 2:** Morphometric mean values of *lapilli*, standard deviation (s.d.) and minimums (Min.) and maximus (Max.) values divided for the size classes investigated: Maximum otolith length (OL, mm), the maximum otolith width (OW, mm), otolith perimeter (OP, mm) and otolith surface (OS, mm^2^), the ratio of otolith length to the total fish length (OL/TL), circularity (C = OP^2^/OS), rectangularity (Re = OS/[OL×OW]), ellipticity (E = OL–OW/OL+OW), aspect ratio (AR = OW/OL%), form factor (FF = 4πOS/OP^2^) and roundness (Ro = 4OS/πOL^2^).

	CLASS I			CLASS II			CLASS III			CLASS IV		
	Mean	s.d.	Min.—Max.	Mean	s.d.	Min.—Max.	Mean	s.d.	Min.—Max.	Mean	s.d.	Min.—Max.
**OL**	0.178	0.014	0.158–0.204	0.224	0.012	0.193–0.249	0.285	0.026	0.250–0.318	0.325	0.022	0.293–0.347
**OW**	0.169	0.013	0.146–0.190	0.222	0.019	0.192–0.261	0.292	0.019	0.254–0.326	0.321	0.013	0.303–0.342
**OP**	0.554	0.029	0.489–0.597	0.715	0.045	0.624–0.817	0.935	0.077	0.824–1.048	1.035	0.027	0.992–1.070
**OS**	0.024	0.002	0.018–0.027	0.040	0.004	0.030–0.050	0.065	0.009	0.051–0.081	0.078	0.004	0.073–0.084
**OL / TL**	0.010	0.002	0.009–0.017	0.01	0.001	0.006–0.008	0.008	0.001	0.006–0.010	0.007	0.001	0.006–0.008
**C**	12.975	0.133	12.751–13.176	12.989	0.104	12.827–13.180	13.361	0.363	12.899–14.020	13.633	0.243	13.427–14.243
**Re**	0.783	0.019	0.736–0.807	0.790	0.018	0.748–0.824	0.785	0.032	0.728–0.841	0.754	0.017	0.718–0.781
**E**	-0.607	0.120	-0.803–-0.409	-0.547	0.077	-0.703–-0.439	-0.451	0.088	-0.563–-0.307	-0.344	0.113	-0.526–-0.235
**AR**	0.955	0.118	0.772–1.158	0.994	0.086	0.871–1.182	1.029	0.060	0.903–1.105	0.991	0.102	0.893–1.160
**FF**	0.969	0.009	0.954–0.986	0.968	0.007	0.954–0.980	0.942	0.025	0.897–0.975	0.923	0.015	0.883–0.937
**Ro**	1.058	0.135	0.808–1.298	1.018	0.097	0.804–1.167	0.974	0.082	0.888–1.183	0.975	0.090	0.820–1.074

**Fig 6:**
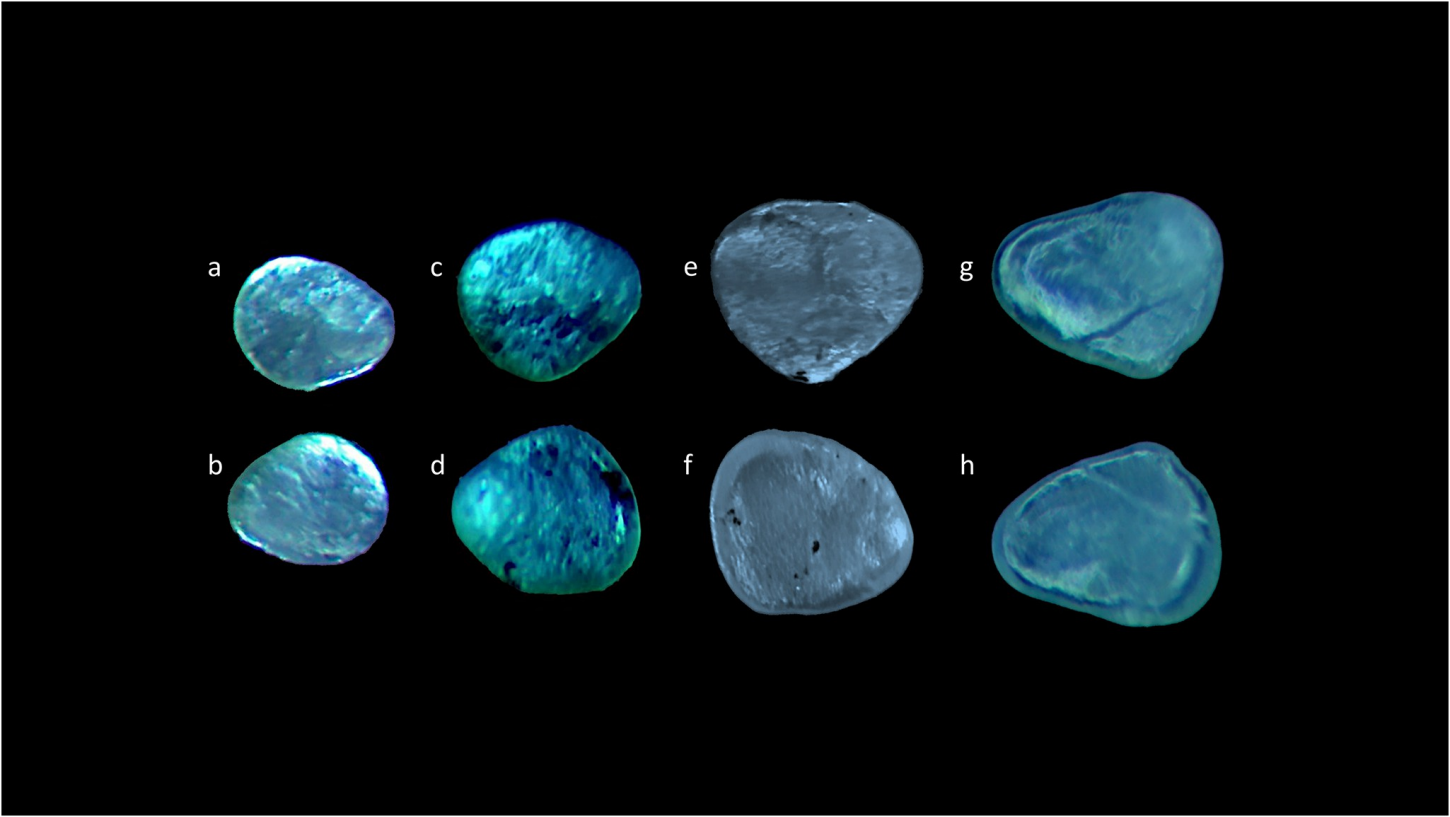
Stereoscope images of left (a,c,e,g) and right (b,d,,f,h) *lapilli* dorsal surfaces belonging to size Classes I (a,b), II (c,d), III (e,f) and IV (g,h).

Concerning the morphological differences between size classes (Fig 6a–6h), specimens belonging to Class I showed *lapilli* with a globular shape characterized by an enhanced asymmetry between internal and external zones. Class II showed a most ovoidal shape with an increased symmetry between internal and external zones. The *extremum posterior* was less triangular in Classes I and II than in Classes III and IV. In Classes III and IV, *lapilli* showed the most irregular shapes, with very prominent *prominentia marginalis* and *extremum posterior*, as also highlighted by the increase of ellipticity (E) value and the decrease of circularity (C) values (Table 2).

ANOVA showed significant differences for almost all the morphometric measurements of the *lapilli* between the four size classes examined (p < 0.05). Some exceptions were found for roundness and OW/OL%, which did not show any significance among any size class (P > 0.05). Form-Factor, Ellipticity and P^2^/A showed no significant changes between Class I and II (p >0.05). Finally, A/(OLxOH) showed no variability between Classes I, II and III (p > 0.05) (S5 Table). A significant correlation between the body weight and total length of the specimens and the *lapilli* morphometries was observed for all parameters except for OW/OL% (P > 0.05) (S6 and S7 Tables). The morphometrical parameters did not show significant differences between the right and left side *lapilli* for each size class investigated (p>0.05).

**Fig 7:**
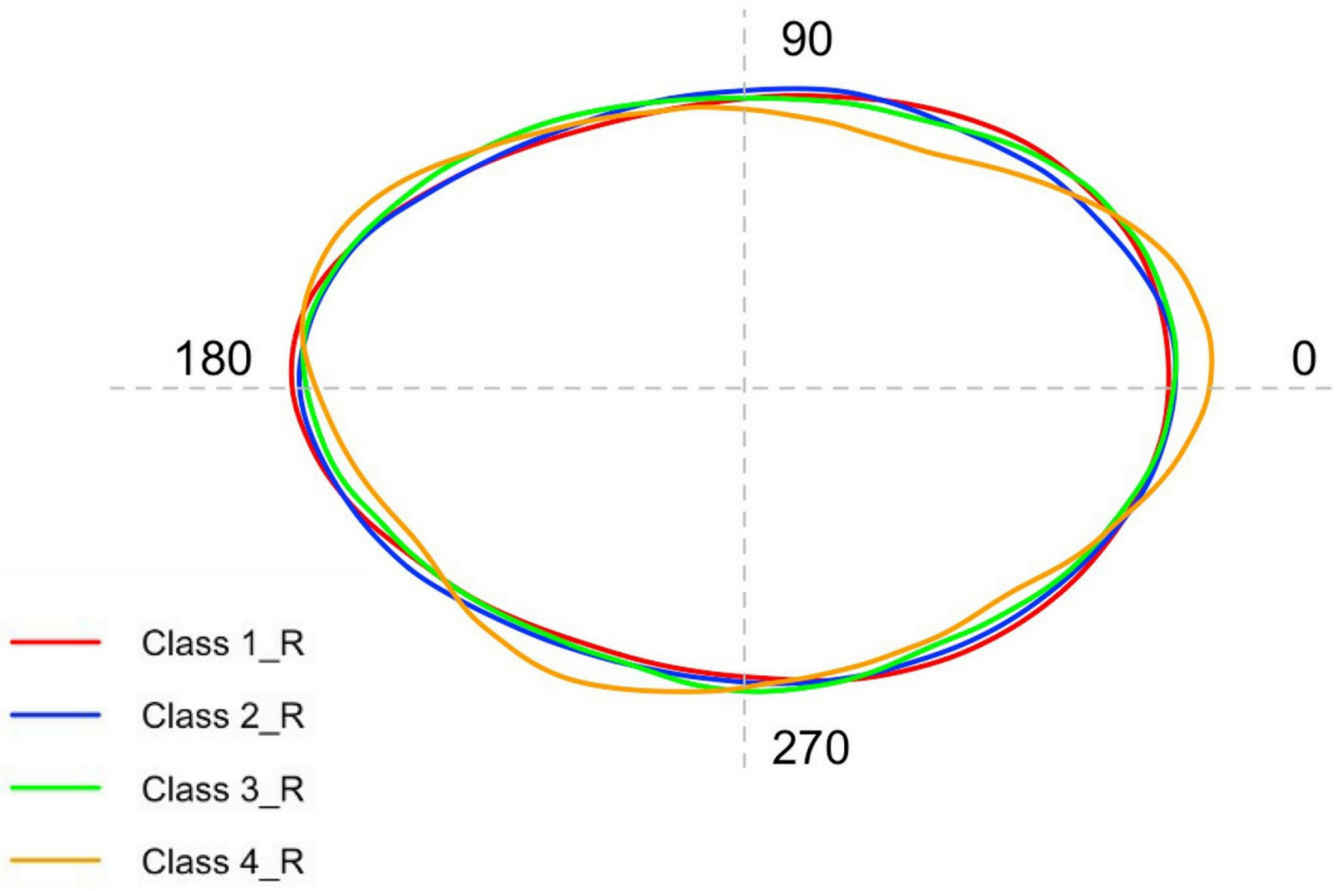
Mean shape of right *lapilli* contours belonging to the four investigated size classes.

The graph in Fig 7 represents the mean otolith shape comparison among different size classes for right *sagittae* obtained through standardized Wavelet coefficients. The quality of both Wavelet and Fourier reconstruction was estimated by comparing the deviations from the otolith outlines, with the value 15 sets as the maximum number of Fourier harmonics to be shown (S1 Fig). The mean and standard deviation of calculated coefficients was plotted using the gplots R package to assess how the variation of Wavelet coefficients depends on the position along the outline (S2 Fig).

**Fig 8:**
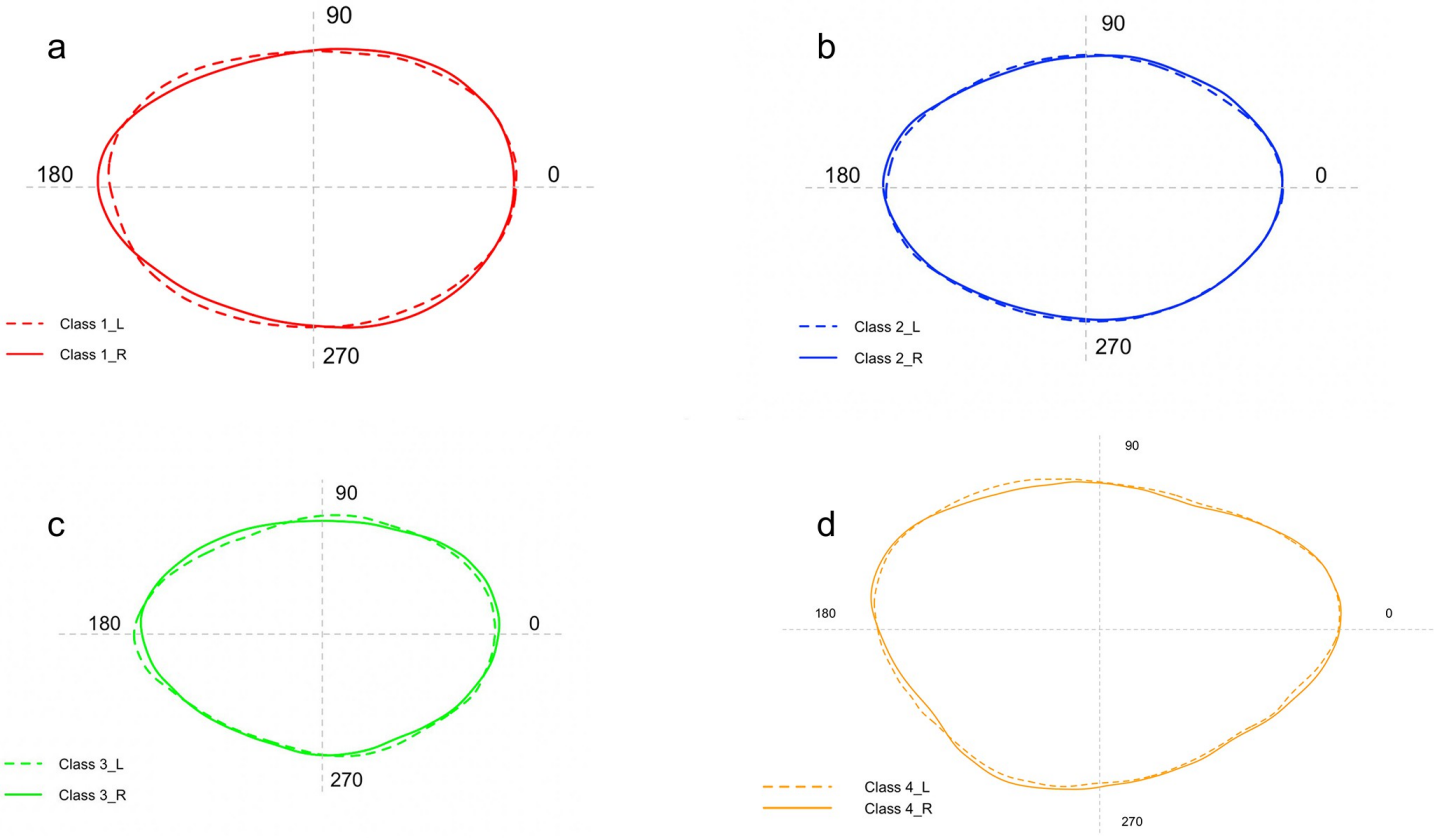
Comparison between the mean shape of left and right *lapilli* contours belonging to Class I (a), Class II (b), Class III (c) and Class (IV).

**Fig 9:**
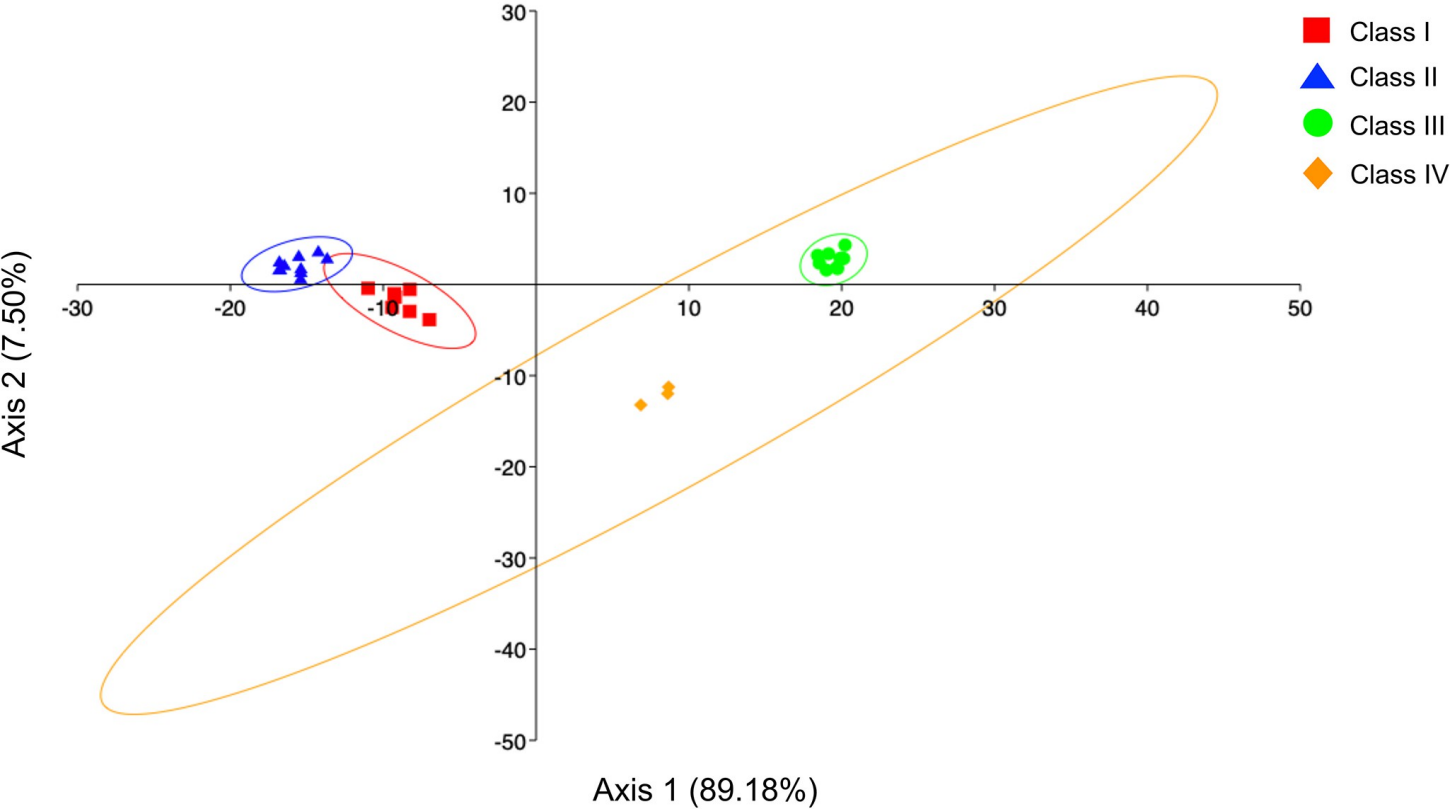
Linear Discriminant Analysis (LDA) computed between the size class analyzed, calculated on Wavelet Fourier descriptors obtained by the left side of *lapilli*. Ellipses includes 95% confidence interval.

The investigations carried out on the shape analysis showed a significant difference between the right and left sides of the *lapilli* only for Class IV (p = 0.017, df = 1, F = 4.12) (Fig 8a–8d). Furthermore, significant variability was observed between size classes for the left side (p = 0.01). This result was confirmed by the LDA, also in agreement with what was obtained from the analysis of variance, highlighting how classes I and II are markedly separated from classes III and IV, as shown in Fig 9 (Axis 1 89.18%).

### Scanning Electron Microscopy (SEM) analysis

**Fig 10:**
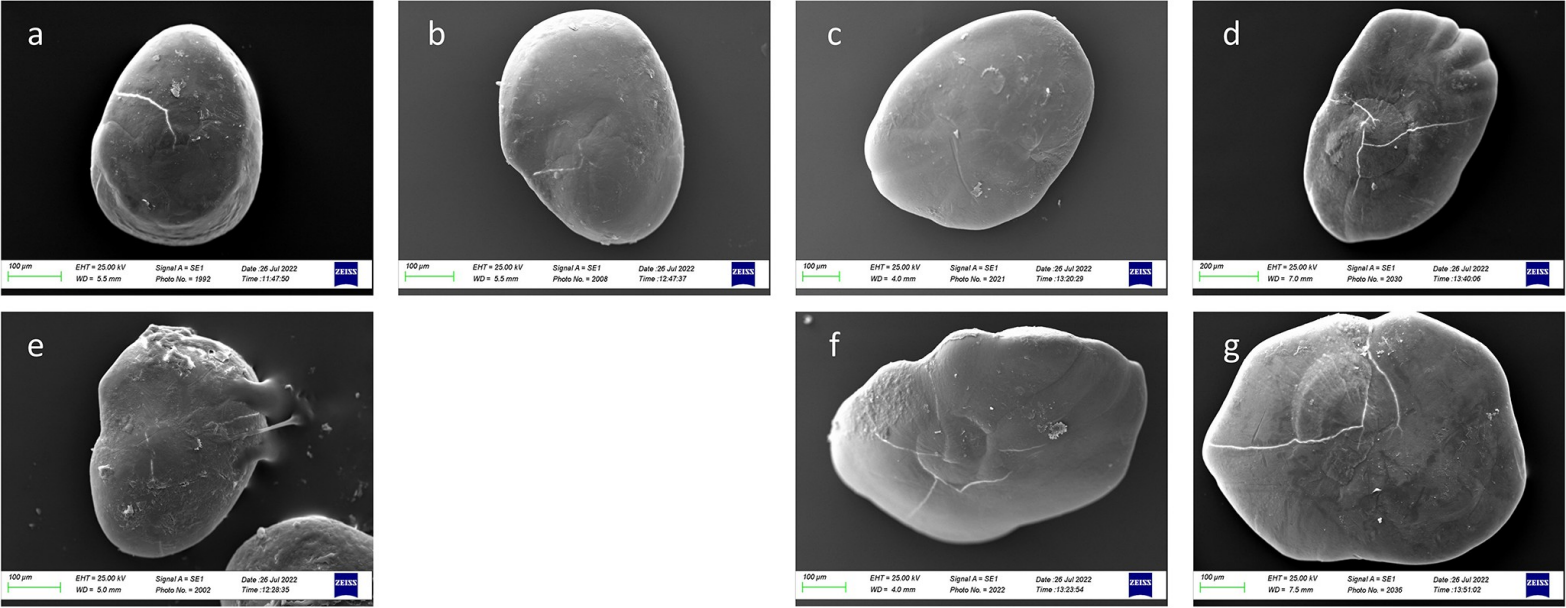
SEM images of *A*. *hemigymnus’ sagittae* inners (a, b, c, e) and external (d, f, g) surfaces separated for size classes: Class I (a, e), Class II (b), Class III (c, f) and Class IV (d, g).

As shown by Fig 10a–10g, the external textural organization of *sagittae* was uniform, with a granular surface and an almost completely homogeneous dimension and orientation of crystals. The *sulcus acusticus* was heterosulcoid, located on the longitudinal midline of the *sagitta* with a bi-ostial opening (Fig 10a–10c and 10e). The *ostium* was deep with a rectangular to funnel-like shape, while the *cauda* was very different, superficial and with a not well-defined ventral limit.

**Fig 11:**
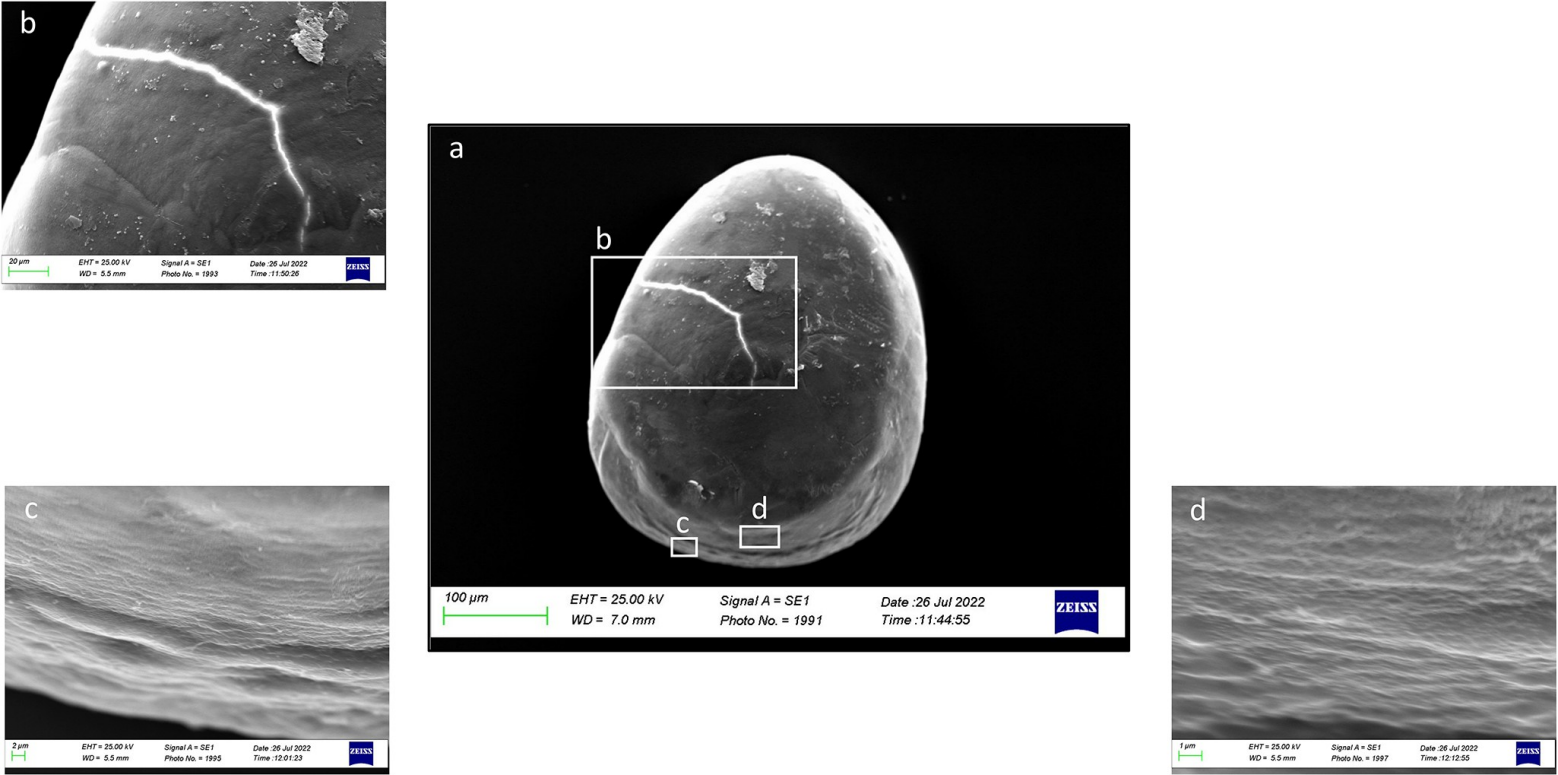
SEM images of a right *sagitta* inner surface belonging to size Class I (a) with details of the external textural organization of *sulcus acusticus* (b) and concentric deposition planes of carbonate detected in ventral margin (c and d).

**Fig 12:**
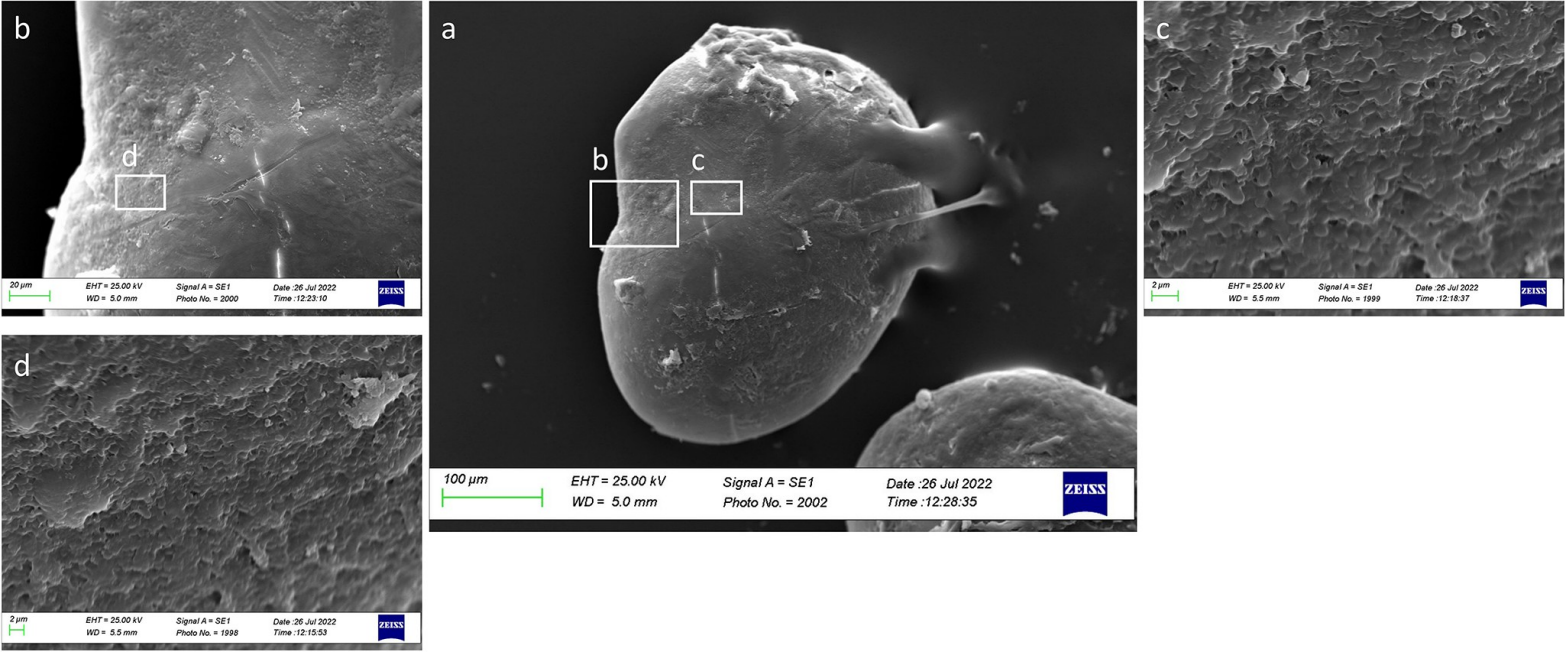
SEM images of a left *sagitta* inner surface belonging to size Class I (a) with detail of *sulcus acusticus* (b), *ostium* (c) and *crista superior* (d) external textural organization.

In Class I, it was visible, at a superficial view, the carbonate daily increments on a concentric deposition plane (Fig 11a, 11c and 11d), which made the ventral margins jagged and irregular. The superficial crystalline habit was uniform, characterized by the presence of small aragonitic crystals with an irregular granular shape, organized in overlapping successive concentric thin layers that made the orange skin-like surface rough (Fig 12a and 12c). As highlighted in Figs 11b, 12b and 12d, crystal regions of various sizes and shapes in the *sulcus* were also detected. The presence of large crystals was detected near the ventral margins and the *crista superior*.

**Fig 13:**
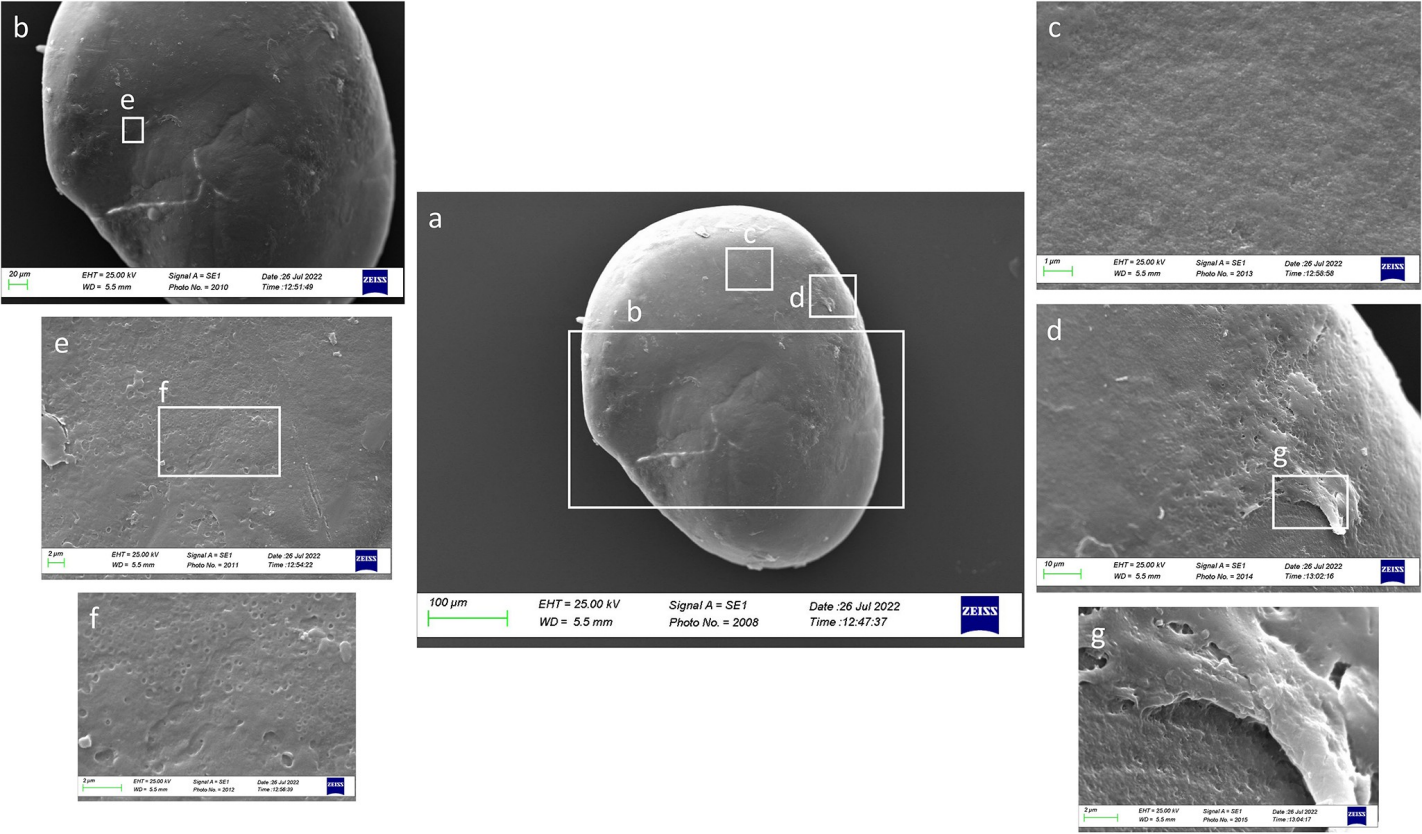
SEM images of a left *sagitta* inner surface belonging to size Class II (a) with details of *sulcus acusticus* (b), with its external textural organization (e, f), and crystalline habits of the posterior otoliths zone (c,d,g).

In Class II, the surface became smoother than in Class I and fine-pored, with the characteristic small aragonitic prismatic crystals with a regular shape and organization (Fig 13a, 13c and 13d). *Sulcus acusticus* became larger without carbonate sculpturing organized in growing units (Fig 13b, 13e and 13f). Some carbonate crystals were associated in lamellae in the posterior otoliths area, forming large superficial wave-like structures (Fig 13g).

**Fig 14:**
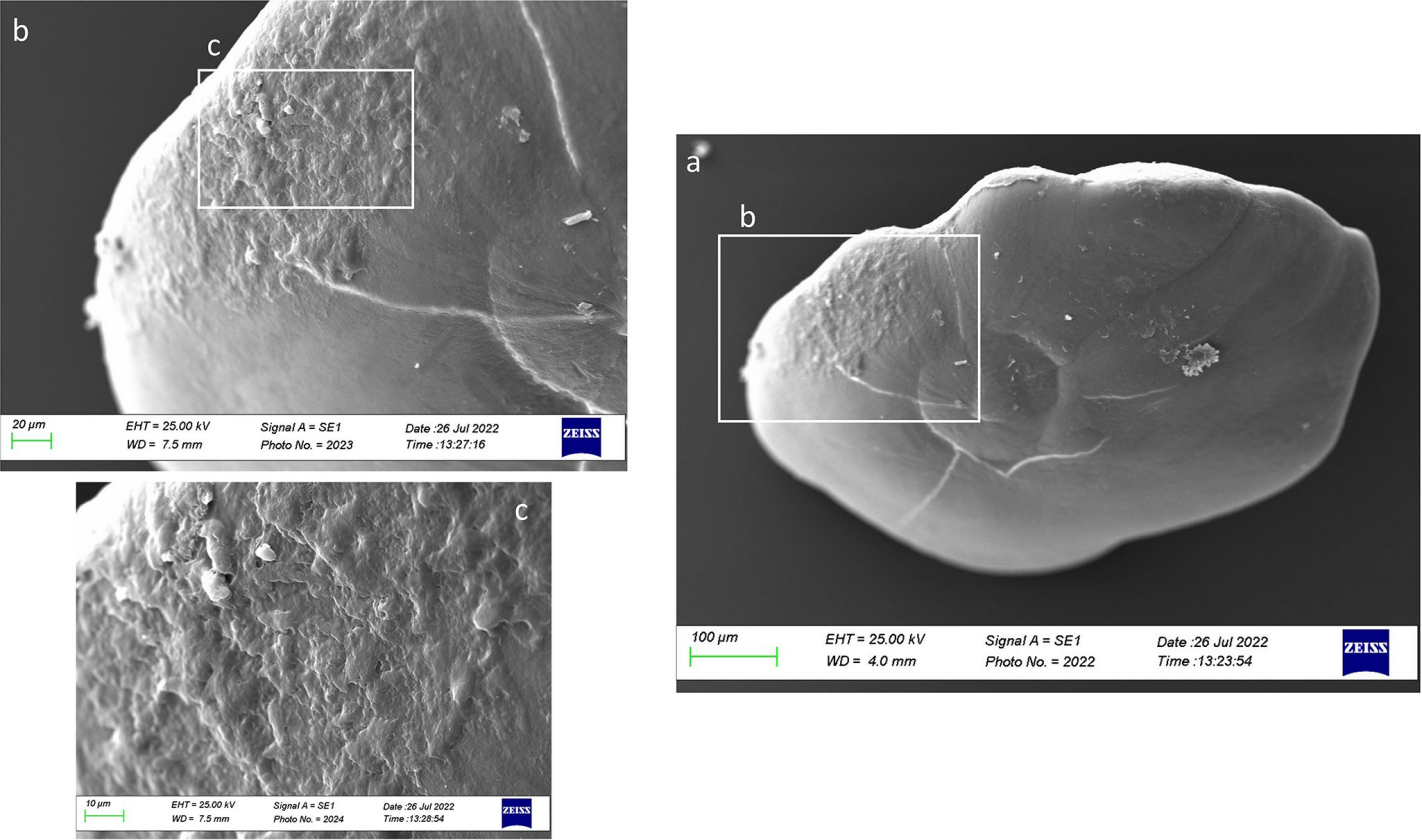
SEM images of a right *sagitta* external surface belonging to size Class III (a) with detail of the external textural organization near the *excisura ostii* (b, c).

In Class III, the surface became more irregular than in the last two classes, with different carbonate polymorphs, characterized by large botryoidal to hexagonal prisms crystals, in the internal surface near the *excisura ostii* (Fig 14a–14c). In the internal face was visible a circular groove (Fig 14a) representing the core of the *sagitta*, characterized by an external crystalline organization uniformly composed of small regular crystals.

**Fig 15:**
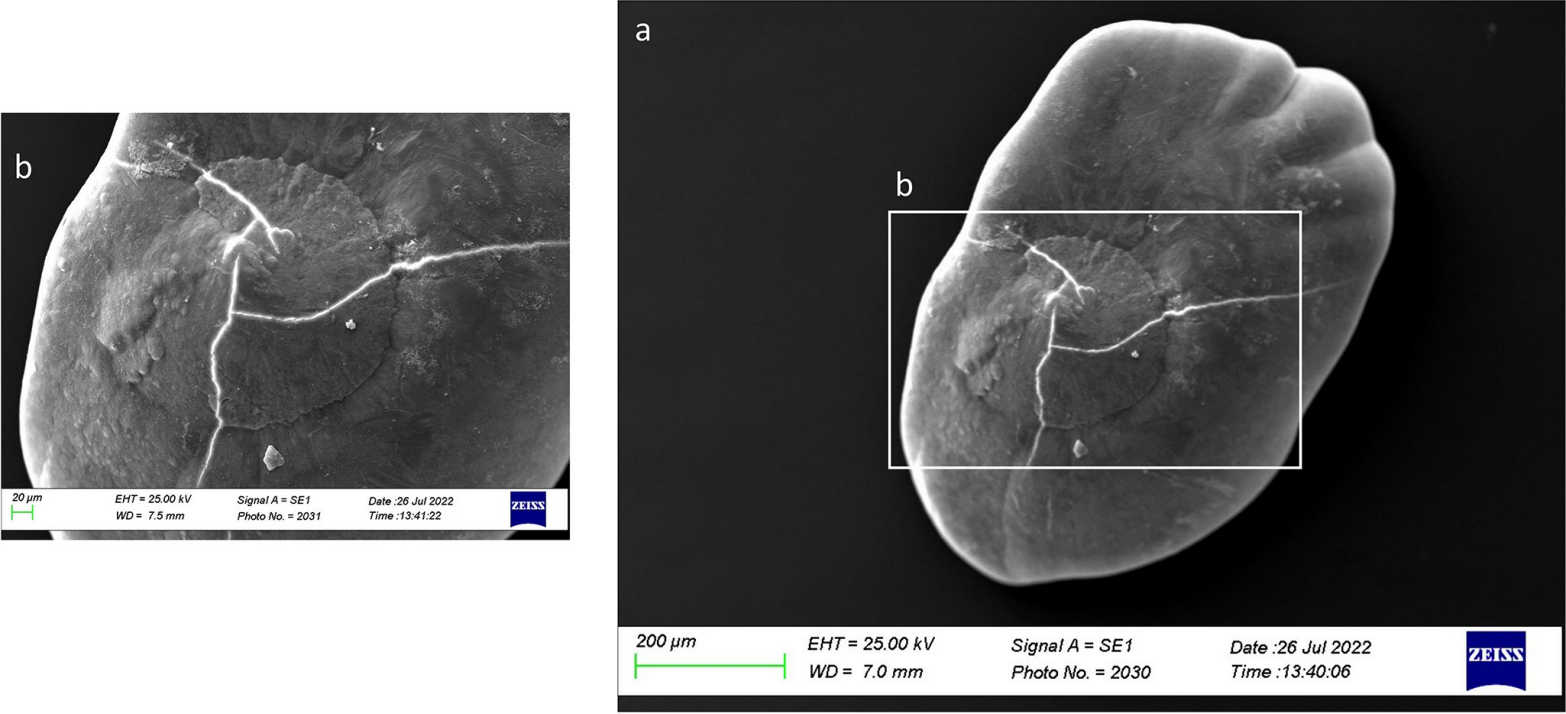
SEM images of a right *sagitta* external surface belonging to size Class IV (a) with details of the external textural organization of the *core* zone (b).

**Fig 16:**
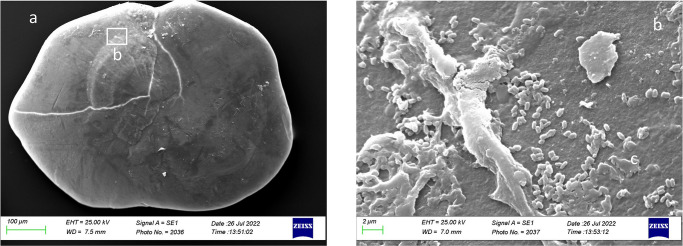
SEM images of a right *sagitta* external surface belonging to size Class IV (a) with details of the crystalline habits and external textural organization (b).

In Class IV, the external textural organization was uniformly characterized by globular secretions widely distributed on the whole otolith surface (Fig 15a and 15b). As shown by Fig 16b, the presence of aragonitic crystals forming superficial wave-like lamellae was also reported. The carbonate crystal habit was mainly composed of aragonitic crystals organized in distinct uniform plates, with large carbonate sculpturing inside the circular groove of the core (Figs 15a and 16a). These made the central *sagitta* zone most irregular than the peripherical ones.

**Fig 17:**
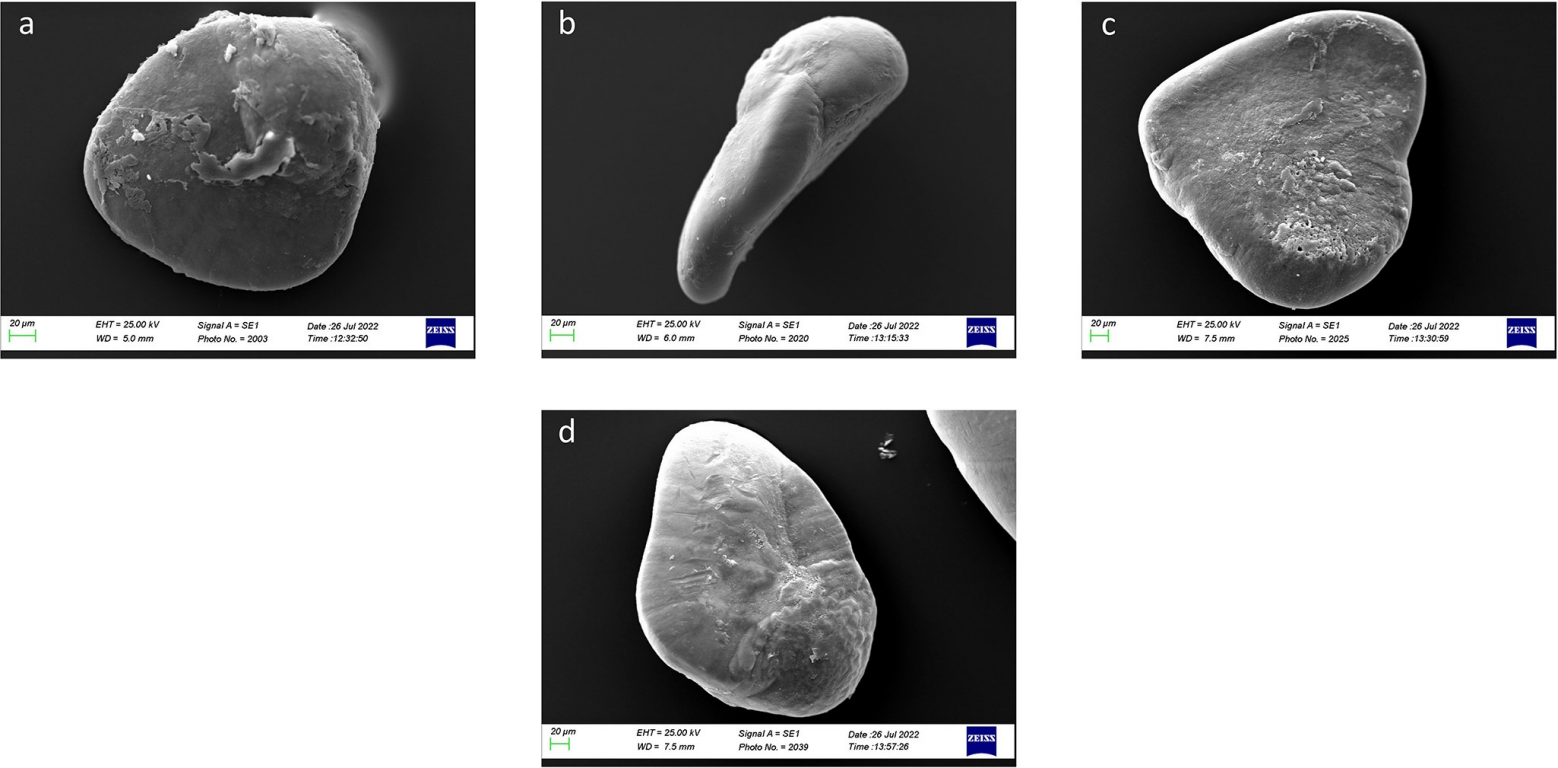
SEM images of ventral (a, d), lateral (b) and dorsal (c) surfaces of *A*. *hemigymnus’ lapilli* separated for size classes: Class I (a), Class II (b), Class III (c) and Class IV (d).

As shown in Fig 17a–17d, the external textural organization of *lapilli* was irregular, with different carbonate polymorphs and crystals with different orientations and sizes. The surface was granular to fine-pored, with edges on the ventral and posterior faces, especially near *confluentia gibbus maculae*. The ventral face was characterized by large crystals, especially on the *gibbus maculae*. *Sulcus lapillus* was thin and superficial, characterized by a uniform orientation of crystals and a regular external textural organization.

**Fig 18:**
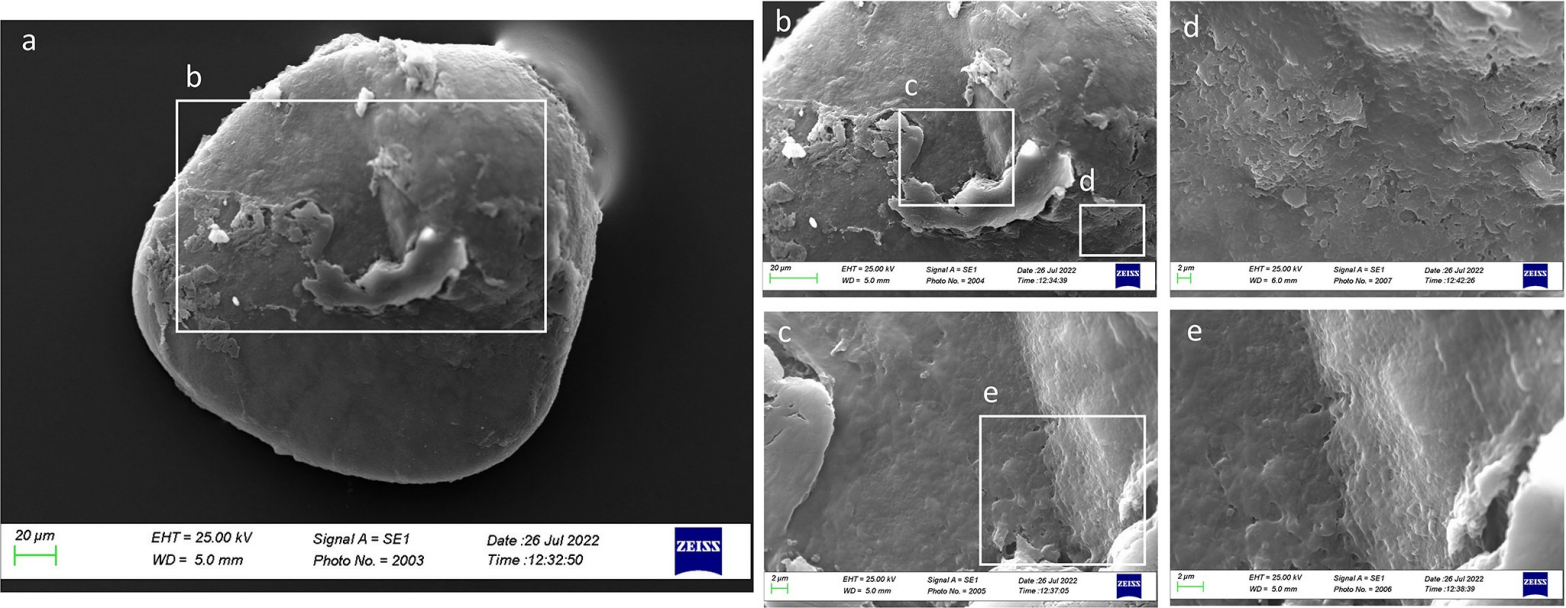
SEM images of a *lapillus* ventral surface belonging to size Class I (a) with details of *sulcus lapillus* (b, c) and *gibbus maculae* (d, e) external textural organization.

In Class I, the ventral face was characterized by different carbonate polymorphs with different orientations. Large rhombohedral and hexagonal crystals were detected on *gibbus maculae* and near the margins of the inner and anterior otoliths (Fig 18a, 18b and 18d). *Sulcus lapillus* showed a different external textural organization and a uniform surface, with small aragonitic crystals with a regular shape and orientation (Fig 18c).

**Fig 19:**
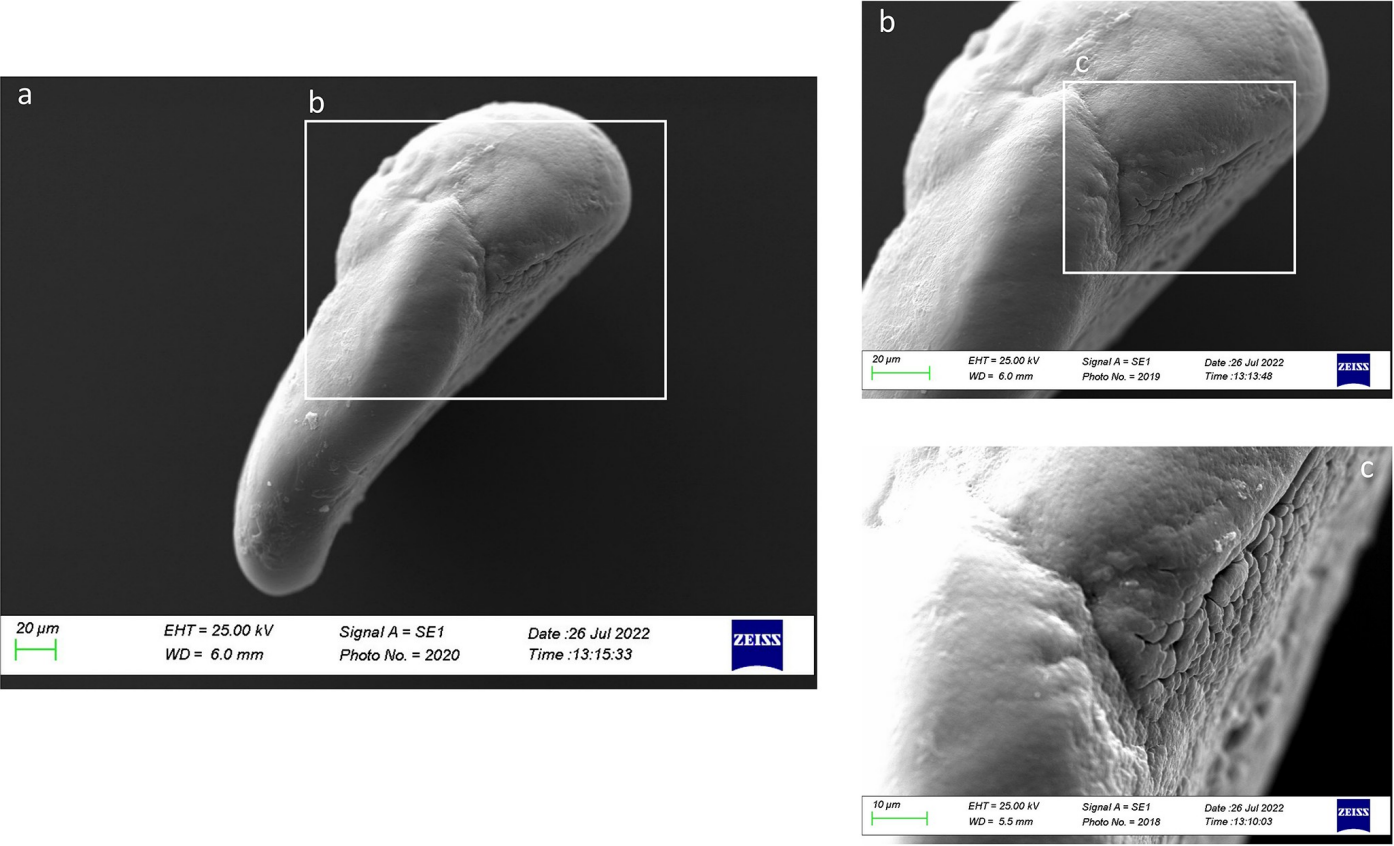
SEM images of a *lapillus* lateral surface belonging to size Class II (a) with details of *gibbus maculae* (b) and *confluentia gibbi maculae* (c) crystalline habits.

From a lateral view (Fig 19a–19c), the *lapillus* of Class II showed an irregular external textural organization, with large rhombohedral crystals on *gibbus maculae* and the *confluentia gibbi maculae*. It was also reported the presence of several edges on the *confluentia gibbi maculae* and the ventral face, separating the large prismatic crystals with a discontinuity in their orientation (Fig 19c).

**Fig 20:**
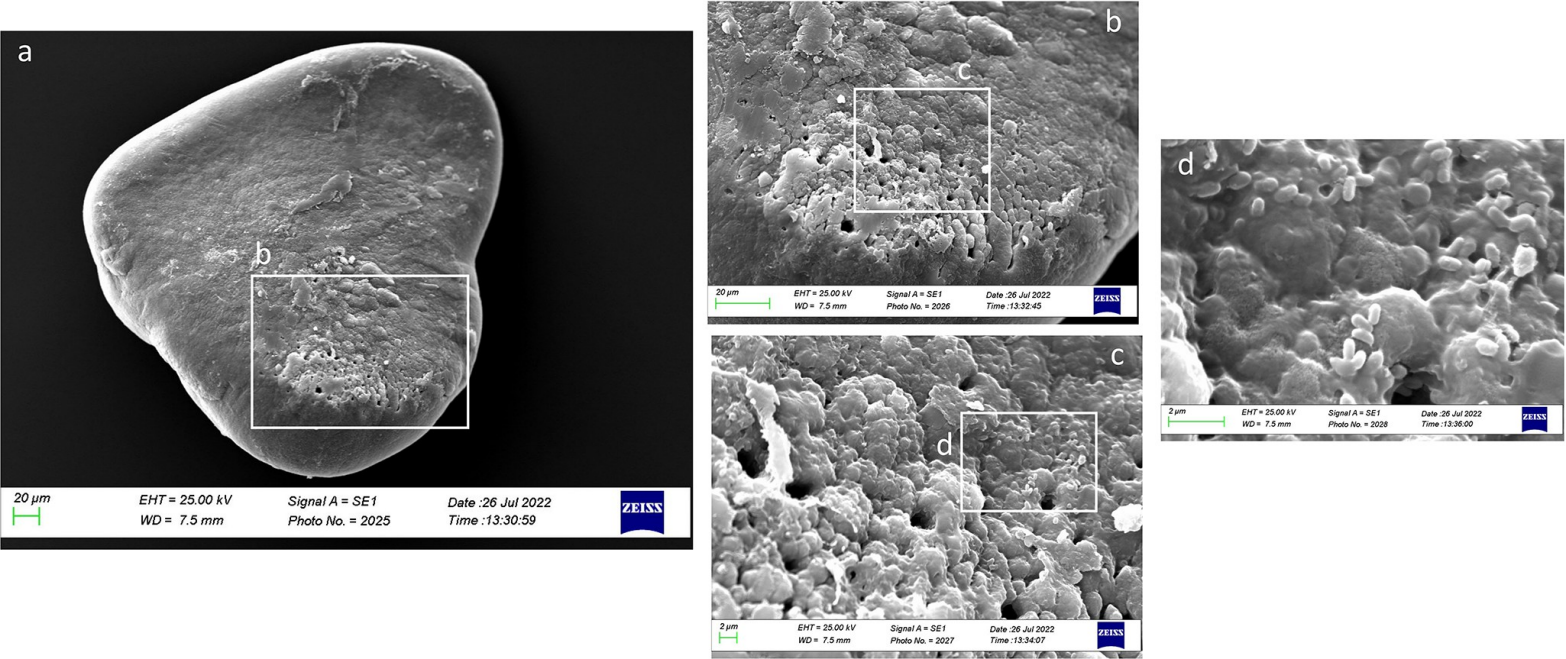
SEM images of a *lapillus* dorsal surface belonging to size Class III (a) with details of the external textural organization near the external margin (b, c).

In Class III, the dorsal face was characterized by large prismatic and hexagonal crystals that alternated with small aragonitic crystals, making the surface irregular (Fig 20a–20c). The presence of globular secretion was also reported often absorbed in the growing otoliths matrix (Fig 20d). Several deep pores were reported near the external margin, separating the large prismatic and hexagonal crystals with different orientations.

**Fig 21:**
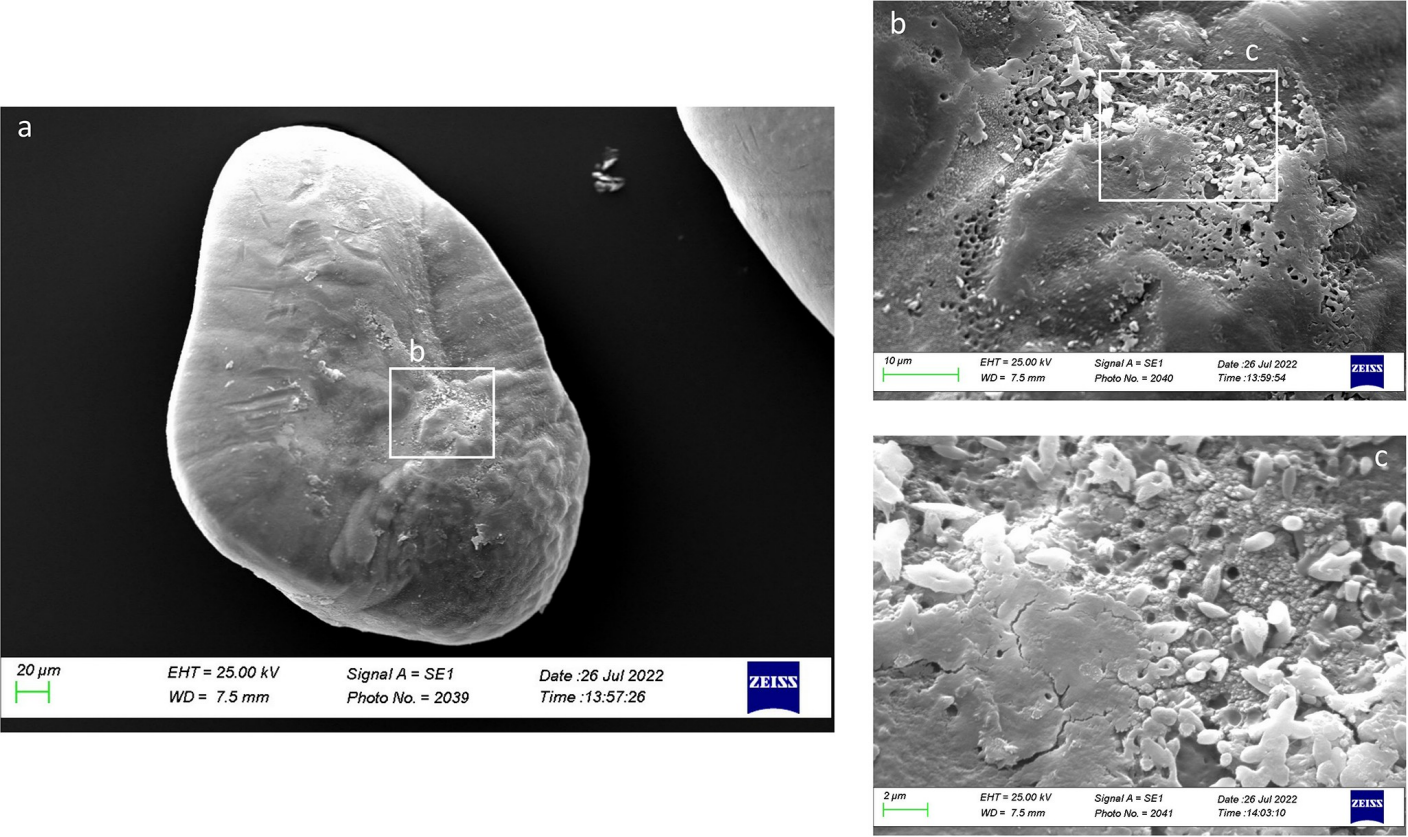
SEM images of a *lapillus* ventral surface belonging to size Class IV (a) with detail of the *gibbus maculae* and *prominentia marginalis* external textural organization and crystalline habits (b, c).

As reported in Fig 21a–21c, in Class IV, the external textural organization was regular, with the presence of prismatic aragonite crystals organized in plates in almost all the otolith’s surface except on the *gibbus maculae* and *prominentia marginalis*, characterized by the presence of large rhombohedral crystals. It was also reported the presence of deep pores separating large plates aggregations of crystals, located on an upper superficial level, from small prismatic carbonates association located on a lower level (Fig 21c).

## Discussion

Improving the knowledge base on otoliths of mesopelagic species is essential to understand their eco-morphological adaptation to deep environments. Indeed, the variability of inner ear morphology among individuals of the same species inhabiting different geographical areas reflects the adaptation capability of marine organisms to environments under different evolutionary pressures. Despite the physiological processes allowing otoliths’ shapes and morphological intraspecific variations at a geographical scale are still now largely unknown, it is widely reported how diet [106], temperature [107], genetic lineage [108], soundscape [109] and physicochemical features of water masses [61] can lead to these differences, impacting hearing ability (in addition to otoliths mass, endolymph viscosity and *sulcus acusticus* dimensions) and shaping otoliths’ contours and morphology. According to this, the present paper confirmed the otoliths variability in populations of the studied species inhabiting different environments in both *lapilli* and *sagittae*. Results showed a morphology of *sagittae* different than those reported in the literature on the same species from other geographical areas [25, 27, 32, 38, 110, 111]. *A*. *hemigymnus* specimens inhabiting the Strait of Messina had larger *sagittae* than those from the western Mediterranean Sea and Atlantic Ocean. This was highlighted by higher OL/TL values, Re and C, than those reported by Tuset in 2008 [25] for individuals with a total length between 30 and 40 mm. The larger surface and perimeter, together with a conformation most squared (than round) of posterior and anterior regions, and most angled to peaked of dorsal and ventral regions, markedly different than those reported in the literature, could be strictly related to the peculiarity of the studied area. The Stait of Messina is characterized by unique oceanographic features [97, 98] that inevitably influence habitats and inhabiting species. The morphological features of *sagittae* shown by results, more similar to those described in specimens from the Portuguese Atlantic Ocean and Northwestern Atlantic Ocean [38, 111] than Mediterranean ones, could be indeed connected with the environmental features of the Strait of Messina. Several external factors, such as those related to the environment (e.g., temperature, pH, salinity, depth) [96, 112–117] or food availability [106], could influence otoliths morphology, shape and morphometry. In this context, the strong tidal currents regime acting in the Strait of Messina, together with the peculiar chemical features of the sea masses, similar in temperature, nutrients, and oxygen concentrations to the Atlantic waters, could induce an otoliths morphology and shape different to those exhibited in species inhabiting other Mediterranean areas. Also, *lapilli* described in the present paper showed a similarity in morphology and morphometry with those described in Atlantic populations by Assis [38], currently the only descriptions reference of *A*. *hemigymnus lapilli* in literature.

For this reason, it was not possible to compare with Mediterranean Sea data. According to the literature [6, 34], otoltihs’ shape, size and morphology, in addition to the dimension and shape of sensory epithelia and ear structure, are intimately related to sound detection, discrimination between sounds with differences in frequencies and/or intensities, determination of sounds’ direction in a three-dimensional space and detecting of sound signals in the presence of unwanted sounds. At the interspecific level, otoliths’ differences in teleosts’ fishes reflect the different hearing abilities that are strictly related to the different life habits, ecology and life history traits of the species. From an intra-specific point of view, otoliths’ shape and morphological differences between the different populations of the same species can be allowed by several factors. Diet influences the composition and quantity of endolymph proteins, which are fundamental for otoliths’ biomineralization processes [118]. For this reason, diet variations between populations of the same species from different geographical areas may induce differences in otolith morphology and shape. Water masses’ physicochemical differences between geographical areas and genetic differences between population lineages can also induce these differences. For example, the temperature can drive growth and morphological variations in deep-sea fishes, as reported for the black spot sea beam *Pagellus bogaraveo*, Brünnich, 1768 [119], while the overall contrasting environmental conditions between different marine areas can influence the overall otoliths morphological and outlines differences, as highlighted for the populations of coral reef snapper *Lutjanus kasmira*, Forsskål, 1775 from the Pacific Ocean [108]. Otherwise, genetic variations at the intra-specific level, such as those derived from long-time separation events among populations, only affect otolith locally, mainly in the rostrum and antirostrum parts [108]. Concerning the present paper, further analysis of the inner ears of species inhabiting the Strait of Messina, comparing the populations from this area with those from others, are required to confirm and better understand the influence of these peculiar environments on marine organisms deepening the knowledge on their eco morphological adaptation.

Concerning the variation in otolith morphology and shape related to specimens’ total length and weight, results showed clear differences between the size classes in both *sagittae* and *lapilli*. The absence of literature data on size-related otoliths variations from other geographical areas on the studied species makes it challenging to investigate the environmental influence on the variation among different size classes. *A*. *hemigymnus* showed an overall morphology and shape of *sagittae*, often widespread in fish inhabiting deep environments which perform simple movements in the water column [6, 120]. Unlike other mesopelagic species, which show elongate *sagittae* with a pronounced *rostrum*, the studied species showed tall *sagittae*, small, with a not pronounced short *rostrum*, like those reported for other Stomiiformes species (e.g., *Chauliodus sloani*, Bloch & Schneider, 1801) [25, 111, 121].

The significant difference in shape, morphometry and morphology showed by results for both *lapilli* and *sagittae* between size classes could be strictly related to the life history and biology of the studied species. Indeed, *A*. *hemigymnus* is a mesopelagic predator with a relatively high trophic level, mainly hunting on zooplankton (e.g., chaetognaths, euphausiids, copepods), fish and gelatinous plankton. The feeding habits of this species varies geographically according to prey availability and distribution, showing a niche and resource partitioning with the other mesopelagic predators [47, 89, 92, 93]. The vertical distribution of this species also shows geographical variations mainly related to water temperature, with the deepest distribution reported in oceanic populations than in the Mediterranean ones, which show mostly enhanced migratory behaviour. Indeed, the daily vertical migrations performed by this species are widely reported in the Mediterranean Sea [47, 93, 122]. Also, individuals’ size and ontogenetic stages influence their vertical distribution, migratory behaviour and diet, with small specimens that do not perform large vertical movements, inhabit shallower depths than larger ones, and mainly prey on small copepods [123, 124]. These differences in life and feeding habits could influence the inner ear structures and morphology in the different size classes. Indeed, *lapilli* and *sagittae* showed significant differences for all the morphometrical indices between all the size Classes. The larger *sagittae*, with a bigger surface and increased rectangularity and roundness, reported for individuals belonging to size Classes III and IV, could be related to the variation in life habits and ecology during their growing process. Indeed, as widely reported in the literature, many free and fast swimming fishes, which perform large movements, are characterized by large sagittal otoliths, with enhanced dorsal and ventral regions characterized by large *crista superior* and *inferior* [114, 115, 120, 125, 126]. The variations reported by results for *sagittae* between the different size Classes could be in line with these ecomorphological features. Concerning *lapilli*, they also showed clear differences in shape, morphology, and morphometry between size Classes. According to the literature [35, 38], *lapilli* have a most regular intra and inter-specific morphology if compared to *sagittae*. As shown by the results, the *lapilli* of *A*. *hemigymnus* had a circular morphology and shape maintained in all the size classes. The pointed and most pronounced *extremum posterior*, with a triangular shape, in individuals belonging to Class III and IV than those of other classes has led to significant differences reported for morphology and shape. The lack of reference data from the literature on utricular otoliths did not allow for an ecomorphological interpretation of the differences related to specimens’ size. However, as reported for *sagittae*, the variation among size classes could also be related to the studied species’ life history. Further analysis on ontogenetic and size-related variations on sagittal and utricular otoliths features, biology and ecology of mesopelagic species are required to understand the relation between inner ears eco morphological adaptation and life history traits of these species.

Regarding the differences between left and right sagittal and utricular otoliths, results showed significant differences only regarding otoliths contours. *A*. *hemigymnus* population from the Strait of Messina showed a fluctuating asymmetry [94, 127] in *sagittae*, considered as the presence of random deviations from the perfect symmetry between left and right otoliths. *Lapilli* showed a more marked bilateral symmetry, with the asymmetry reported only for Class IV, confirming their most enhanced intraspecific stability than *sagittae*. The fluctuating asymmetry between otolith pairs can be related to environmental heterogeneity or stress [128, 129]. The studied area is characterized by large fluctuations of oceanographic features related to the strong tidal currents’ regime acting in the entire area. Moreover, the vertical movements performed by the studied species can increase the environmental factors’ heterogeneity to which individuals can be exposed. This strong oceanographic instability could allow the asymmetry shown by the results. Indeed, several authors have related the fluctuating asymmetry to a sub-optimally fish growing under stressful conditions or environmental stressors [130]. Concerning the studied area, its oceanographic features allow a substantial water column variability which could induce the fluctuating asymmetry showed by results, as reported in other mesopelagic species (e.g., larvae of *Maurolicus parvipinnis*, Vailliant, 1888 from southern Patagonia) [61]. Further analyses comparing population of *A*. *hemigymnus* from the studied area with others from other habitats are required to confirm the relationship between asymmetry and environmental heterogeneity of the studied area.

The overall uniform external textural organization of *sagittae* showed by SEM analysis, characterized by the presence of regular small aragonitic crystals with a uniform orientation that made the surface granular, was different from those reported in the literature for other species inhabiting shallower environments [23, 24]. This difference could be related to the stability of mesopelagic strata. Indeed, deep marine environments did not show large fluctuations in chemical and oceanographic features. According to the literature, large crystals with chaotic orientation can be associated with physiological stress and environmental instability [131–134]. Moreover, as reported for a particular ecotype of *Poecilia mexicana*, Steindachner, 1863, inhabiting low-light environments [117], the presence of crystal regions in the *sulcus acusticus* characterized by various sizes and shapes could be related to the low light arriving in the mesopelagic environment. Comparing results from the present paper with those of literature on other mesopelagic species, *A*. *hemigymnus* specimens showed a more uniform crystals organization in the *sulcus acusticus* than those reported by Lombarte et al. for species belonging to *Coelorinchus* genus from the Southeast Atlantic [135]. The presence of large-size crystal aggregates characterized these last. In contrast, in the studied species, the large crystals in the *sulcus* were isolated and surrounded by small aragonitic crystals with the same orientation. This difference could be related to the different feeding habits of the two species (both predators, but the first is specialized in fishes hunting while the second is a zoo-planktivorous species), added to the different environments with different chemical features inhabited by them (Atlantic Ocean and Strait of Messina). Also, the large crystals detected in other *sagittae* areas (near the ventral margin and the *crista superior*) were isolated, with botryoidal habits, similar to the vateritic crystals showed in literature for *Macruronus novaezelandiae*, Hector, 1871 [1313]. Also, the complex crystalline habits, with a peculiar wave-like macrostructure, showed in some specimens belonging to class II and IV, were reported in vateritic otoliths belonging to *Notothenia microlepidota*, Hutton, 1875 [131]. The presence of botryoidal crystals increased of Class III and IV large specimens, characterized by the entire otoliths zone of the inner face. The formation of vateritic crystals could be strictly related to transient ambient events. As stated by Pach et al. [136], temperature shocks and variations of endolymphatic fluid viscosity can induce vaterite deposition. In this context, the vertical migrations performed by large *A*. *hemigymnus* specimens could induce the variations in carbonate polymorphs detected by SEM analysis. Indeed, the movements towards superficial marine strata of this species cause the transition from deep water masses to surficial ones, characterized by different chemical features (e.g., temperature, salinity, oxygen concentration), allowing the possible incidence of transient ambient events. The *lapilli’s* external textural organization showed a more pronounced irregularity than *sagittae* in all the analyzed size classes, with large crystals on *gibbus maculae* and *prominentia marginalis* characterized by shapes from rhombohedral to botryoidal and deep pores and edges on both dorsal and ventral faces. The lack of literature regarding crystal habits and external textural organization of *lapilli* makes it challenging to compare with other species to understand the possible reasons for their crystalline peculiarity. Despite this, results on *lapilli* were in line with those shown by *sagittae*, with an external textural organization which became most complex, with several carbonate polymorphs and textural irregularities, in specimens belonging to class III and IV than in the others. As stated above for *sagittae*, *lapilli’s* external textural organization and crystalline habits could also confirm the influence of migratory habits on carbonate deposition and otoliths’ crystalline structure. Further analyses of the crystalline habits and carbonate polymorphs of otoliths, both utricular and sagittal, of *A*. *hemigymnus* and other mesopelagic species are required to confirm the influence of life habits on the external textural organization of *sagittae*, performing x-ray diffraction and other techniques to detect their polymorphs percentage composition.

## Conclusion

To our best knowledge, concerning *A*. *hemigymnus*, the present paper represents: (i) the first description of *lapilli* from a Mediterranean area, (ii) the first description of otoliths (both utricular and saccular) using shape and SEM analysis and (iii) the first investigation on their intraspecific variability. Results showed a different morphology of *sagittae* compared to data present in literature from other Mediterranean geographical areas, confirming the high intraspecific variability of saccular otoliths between individuals inhabiting different habitats. SEM analysis has provided the first investigation of external textural organization and crystalline habits in *sagittae* and *lapilli* of the studied species, giving information on their superficial structure and morphology, useful to improve the knowledge base on mesopelagic teleost’s adaptation to deep marine environments. Indeed, these new data added to those on otoliths variation related to fish size and fluctuating asymmetry of *sagittae* give a pool of information essential to understand how a peculiar environment, such as the Strait of Messina, can shape the organisms that inhabit it. Further analyses comparing populations from the studied area with those from other geographical areas are required to understand the ecomorphological adaptation of *A*. *hemigymnus* inner ears, showing how different environments and habitats can shape otoliths’ features.

## Supporting information

S1 Table Results of the ANOVA conducted on the morphometric parameters of *sagittae* extracted from specimens of *Argyropelecus hemigymnus* belonging to 4 size classes. (DOCX)

S2 Table Results of correlation analysis between *sagittae* parameters and sample body weight. (DOCX)

S3 Table Results of correlation analysis between sagittae parameters and sample total length. (DOCX)

S4 Table Results of the ANOVA conducted on Wavelet Fourier descriptors obtained by the right side of *sagittae* extracted from specimens of *Argyropelecus hemigymnus* belonging to 4 size classes. (DOCX)

S5 Table Results of the ANOVA conducted on the morphometric parameters of *lapilli* extracted from specimens of *Argyropelecus hemigymnus* belonging to 4 size classes. (DOCX)

S6 Table Results of correlation analysis between l*apilli* parameters and sample body weight. (DOCX)

S7 Table Results of correlation analysis between *lapilli* parameters and sample total length. (DOCX)

S1 Fig Plotting the quality of *sagittae* (a) and l*apilli* (b) outline reconstruction based on Wavelet and Fourier coefficients. The red lines indicate the level of Wavelet and number of Fourier harmonics needed for a 98.5% accuracy of the remodelling.
(TIF)

S2 Fig Mean and standard deviation (sd) of Wavelet coefficients for all combined *sagittae* (a) and *lapilli* (b) and the proportion of variance among size classes (black line). The horizontal axis shows angle in degrees (°) based on the polar coordinates of the mean otoliths shape plot. The centroid of the otolith is the center point of polar coordinates.
(TIF)
