# Supplementary material for: Intraspecific variability of the saccular and utricular otoliths of the hatchetfish Argyropelecus hemigymnus (Cocco, 1829) from the Strait of Messina (Central Mediterranean Sea)
Source: PLoS One. 2023 Feb 14;18(2):e0281621. doi: 10.1371/journal.pone.0281621 (PMC9928127; doi:10.1371/journal.pone.0281621)
Supplement: S1 Table — (DOCX) [file pone.0281621.s001.docx]

|  |  |  |  |  |  |
| --- | --- | --- | --- | --- | --- |
| **Tukey's multiple comparisons test** | **Mean Diff,** | **95,00% CI of diff,** | **Significant?** | **Summary** | **Adjusted P Value** |
| Area Class I vs. Area Class II | -0,07078 | -0,09570 to -0,04587 | Yes | **** | <0,0001 |
| Area Class I vs. Area Class III | -0,1936 | -0,2183 to -0,1689 | Yes | **** | <0,0001 |
| Area Class I vs. Area Class IV | -0,2833 | -0,3086 to -0,2580 | Yes | **** | <0,0001 |
| Area Class II vs. Area Class III | -0,1228 | -0,1451 to -0,1005 | Yes | **** | <0,0001 |
| Area Class II vs. Area Class IV | -0,2125 | -0,2355 to -0,1896 | Yes | **** | <0,0001 |
| Area Class III vs. Area Class IV | -0,08971 | -0,1125 to -0,06698 | Yes | **** | <0,0001 |
| Length Class I vs. Length Class II | -0,08567 | -0,1191 to -0,05229 | Yes | **** | <0,0001 |
| Length Class I vs. Length Class III | -0,4094 | -0,4425 to -0,3762 | Yes | **** | <0,0001 |
| Length Class I vs. Length Class IV | -0,5377 | -0,5716 to -0,5038 | Yes | **** | <0,0001 |
| Length Class II vs. Length Class III | -0,3237 | -0,3536 to -0,2938 | Yes | **** | <0,0001 |
| Length Class II vs. Length Class IV | -0,452 | -0,4828 to -0,4213 | Yes | **** | <0,0001 |
| Length Class III vs. Length Class IV | -0,1283 | -0,1588 to -0,09786 | Yes | **** | <0,0001 |
| Width Class I vs. Width Class II | -0,1387 | -0,1702 to -0,1073 | Yes | **** | <0,0001 |
| Width Class I vs. Width Class III | -0,1405 | -0,1717 to -0,1093 | Yes | **** | <0,0001 |
| Width Class I vs. Width Class IV | -0,1917 | -0,2236 to -0,1597 | Yes | **** | <0,0001 |
| Width Class II vs. Width Class III | -0,001787 | -0,02997 to 0,02640 | No | ns | 0,9984 |
| Width Class II vs. Width Class IV | -0,05295 | -0,08189 to -0,02401 | Yes | **** | <0,0001 |
| Width Class III vs. Width Class IV | -0,05116 | -0,07987 to -0,02245 | Yes | **** | <0,0001 |
| Perimeter Class I vs. Perimeter Class II | -0,4953 | -0,6173 to -0,3734 | Yes | **** | <0,0001 |
| Perimeter Class I vs. Perimeter Class III | -0,9076 | -1,029 to -0,7865 | Yes | **** | <0,0001 |
| Perimeter Class I vs. Perimeter Class IV | -1,313 | -1,437 to -1,189 | Yes | **** | <0,0001 |
| Perimeter Class II vs. Perimeter Class III | -0,4123 | -0,5216 to -0,3030 | Yes | **** | <0,0001 |
| Perimeter Class II vs. Perimeter Class IV | -0,8176 | -0,9298 to -0,7054 | Yes | **** | <0,0001 |
| Perimeter Class III vs. Perimeter Class IV | -0,4053 | -0,5166 to -0,2940 | Yes | **** | <0,0001 |
| Roundness Class I vs. Roundness Class II | 0,03537 | -0,02438 to 0,09512 | No | ns | 0,414 |
| Roundness Class I vs. Roundness Class III | -0,5414 | -0,6007 to -0,4821 | Yes | **** | <0,0001 |
| Roundness Class I vs. Roundness Class IV | -0,6617 | -0,7223 to -0,6010 | Yes | **** | <0,0001 |
| Roundness Class II vs. Roundness Class III | -0,5768 | -0,6303 to -0,5233 | Yes | **** | <0,0001 |
| Roundness Class II vs. Roundness Class IV | -0,697 | -0,7520 to -0,6421 | Yes | **** | <0,0001 |
| Roundness Class III vs. Roundness Class IV | -0,1202 | -0,1748 to -0,06571 | Yes | **** | <0,0001 |
| Form-Factor Class I vs. Form-Factor Class II | 0,05647 | 0,01955 to 0,09340 | Yes | *** | 0,0007 |
| Form-Factor Class I vs. Form-Factor Class III | -0,04086 | -0,07754 to -0,004188 | Yes | * | 0,0227 |
| Form-Factor Class I vs. Form-Factor Class IV | 0,006838 | -0,03064 to 0,04432 | No | ns | 0,9641 |
| Form-Factor Class II vs. Form-Factor Class III | -0,09734 | -0,1304 to -0,06425 | Yes | **** | <0,0001 |
| Form-Factor Class II vs. Form-Factor Class IV | -0,04963 | -0,08361 to -0,01566 | Yes | ** | 0,0013 |
| Form-Factor Class III vs. Form-Factor Class IV | 0,0477 | 0,01400 to 0,08140 | Yes | ** | 0,002 |
| Ellipticity Class I vs. Ellipticity Class II | -0,1411 | -0,2123 to -0,06982 | Yes | **** | <0,0001 |
| Ellipticity Class I vs. Ellipticity Class III | -1,055 | -1,126 to -0,9840 | Yes | **** | <0,0001 |
| Ellipticity Class I vs. Ellipticity Class IV | -1,284 | -1,356 to -1,211 | Yes | **** | <0,0001 |
| Ellipticity Class II vs. Ellipticity Class III | -0,9137 | -0,9775 to -0,8498 | Yes | **** | <0,0001 |
| Ellipticity Class II vs. Ellipticity Class IV | -1,143 | -1,208 to -1,077 | Yes | **** | <0,0001 |
| Ellipticity Class III vs. Ellipticity Class IV | -0,229 | -0,2940 to -0,1639 | Yes | **** | <0,0001 |
| P^2^/A Class I vs. P^2^/A Class II | -1,632 | -2,649 to -0,6146 | Yes | *** | 0,0003 |
| P^2^/A Class I vs. P^2^/A Class III | 1,005 | -0,005060 to 2,015 | No | ns | 0,0517 |
| P^2^/A Class I vs. P^2^/A Class IV | -0,1366 | -1,169 to 0,8959 | No | ns | 0,9857 |
| P^2^/A Class II vs. P^2^/A Class III | 2,637 | 1,726 to 3,548 | Yes | **** | <0,0001 |
| P^2^/A Class II vs. P^2^/A Class IV | 1,495 | 0,5594 to 2,431 | Yes | *** | 0,0004 |
| P^2^/A Class III vs. P^2^/A Class IV | -1,142 | -2,070 to -0,2134 | Yes | ** | 0,0094 |
| A/(OLxOH) Class I vs. A/(OLxOH) Class II | -0,01484 | -0,02855 to -0,001121 | Yes | * | 0,0286 |
| A/(OLxOH) Class I vs. A/(OLxOH) Class III | 0,01133 | -0,002289 to 0,02496 | No | ns | 0,1378 |
| A/(OLxOH) Class I vs. A/(OLxOH) Class IV | -0,001515 | -0,01544 to 0,01241 | No | ns | 0,992 |
| A/(OLxOH) Class II vs. A/(OLxOH) Class III | 0,02617 | 0,01388 to 0,03846 | Yes | **** | <0,0001 |
| A/(OLxOH) Class II vs. A/(OLxOH) Class IV | 0,01332 | 0,0007022 to 0,02594 | Yes | * | 0,0343 |
| A/(OLxOH) Class III vs. A/(OLxOH) Class IV | -0,01285 | -0,02537 to -0,0003302 | Yes | * | 0,0419 |
| OW/OL % Class I vs. OW/OL % Class II | -0,0833 | -0,1244 to -0,04219 | Yes | **** | <0,0001 |
| OW/OL % Class I vs. OW/OL % Class III | 0,5049 | 0,4641 to 0,5457 | Yes | **** | <0,0001 |
| OW/OL % Class I vs. OW/OL % Class IV | 0,5543 | 0,5126 to 0,5961 | Yes | **** | <0,0001 |
| OW/OL % Class II vs. OW/OL % Class III | 0,5882 | 0,5514 to 0,6250 | Yes | **** | <0,0001 |
| OW/OL % Class II vs. OW/OL % Class IV | 0,6376 | 0,5998 to 0,6755 | Yes | **** | <0,0001 |
| OW/OL % Class III vs. OW/OL %Class IV | 0,04946 | 0,01194 to 0,08698 | Yes | ** | 0,0046 |
| OL/TL Class I vs. OL/TL Class II | -8,497 | -10,03 to -6,962 | Yes | **** | <0,0001 |
| OL/TL Class I vs. OL/TL Class III | -19,28 | -20,80 to -17,75 | Yes | **** | <0,0001 |
| OL/TL Class I vs. OL/TL Class IV | -27,96 | -29,52 to -26,40 | Yes | **** | <0,0001 |
| OL/TL Class II vs. OL/TL Class III | -10,78 | -12,15 to -9,403 | Yes | **** | <0,0001 |
| OL/TL Class II vs. OL/TL Class IV | -19,46 | -20,88 to -18,05 | Yes | **** | <0,0001 |
| OL/TL Class III vs. OL/TL Class IV | -8,683 | -10,08 to -7,281 | Yes | **** | <0,0001 |
| **Tukey's multiple comparisons test** | **Mean Diff,** | **95,00% CI of diff,** | **Significant?** | **Summary** | **Adjusted P Value** |
| Area Class I vs. Area Class II | -0,07078 | -0,09570 to -0,04587 | Yes | **** | <0,0001 |
| Area Class I vs. Area Class III | -0,1936 | -0,2183 to -0,1689 | Yes | **** | <0,0001 |
| Area Class I vs. Area Class IV | -0,2833 | -0,3086 to -0,2580 | Yes | **** | <0,0001 |
| Area Class II vs. Area Class III | -0,1228 | -0,1451 to -0,1005 | Yes | **** | <0,0001 |
| Area Class II vs. Area Class IV | -0,2125 | -0,2355 to -0,1896 | Yes | **** | <0,0001 |
| Area Class III vs. Area Class IV | -0,08971 | -0,1125 to -0,06698 | Yes | **** | <0,0001 |
| Length Class I vs. Length Class II | -0,08567 | -0,1191 to -0,05229 | Yes | **** | <0,0001 |
| Length Class I vs. Length Class III | -0,4094 | -0,4425 to -0,3762 | Yes | **** | <0,0001 |
| Length Class I vs. Length Class IV | -0,5377 | -0,5716 to -0,5038 | Yes | **** | <0,0001 |
| Length Class II vs. Length Class III | -0,3237 | -0,3536 to -0,2938 | Yes | **** | <0,0001 |
| Length Class II vs. Length Class IV | -0,452 | -0,4828 to -0,4213 | Yes | **** | <0,0001 |
| Length Class III vs. Length Class IV | -0,1283 | -0,1588 to -0,09786 | Yes | **** | <0,0001 |
| Width Class I vs. Width Class II | -0,1387 | -0,1702 to -0,1073 | Yes | **** | <0,0001 |
| Width Class I vs. Width Class III | -0,1405 | -0,1717 to -0,1093 | Yes | **** | <0,0001 |
| Width Class I vs. Width Class IV | -0,1917 | -0,2236 to -0,1597 | Yes | **** | <0,0001 |
| Width Class II vs. Width Class III | -0,001787 | -0,02997 to 0,02640 | No | ns | 0,9984 |
| Width Class II vs. Width Class IV | -0,05295 | -0,08189 to -0,02401 | Yes | **** | <0,0001 |
| Width Class III vs. Width Class IV | -0,05116 | -0,07987 to -0,02245 | Yes | **** | <0,0001 |
| Perimeter Class I vs. Perimeter Class II | -0,4953 | -0,6173 to -0,3734 | Yes | **** | <0,0001 |
| Perimeter Class I vs. Perimeter Class III | -0,9076 | -1,029 to -0,7865 | Yes | **** | <0,0001 |
| Perimeter Class I vs. Perimeter Class IV | -1,313 | -1,437 to -1,189 | Yes | **** | <0,0001 |
| Perimeter Class II vs. Perimeter Class III | -0,4123 | -0,5216 to -0,3030 | Yes | **** | <0,0001 |
| Perimeter Class II vs. Perimeter Class IV | -0,8176 | -0,9298 to -0,7054 | Yes | **** | <0,0001 |
| Perimeter Class III vs. Perimeter Class IV | -0,4053 | -0,5166 to -0,2940 | Yes | **** | <0,0001 |
| Roundness Class I vs. Roundness Class II | 0,03537 | -0,02438 to 0,09512 | No | ns | 0,414 |
| Roundness Class I vs. Roundness Class III | -0,5414 | -0,6007 to -0,4821 | Yes | **** | <0,0001 |
| Roundness Class I vs. Roundness Class IV | -0,6617 | -0,7223 to -0,6010 | Yes | **** | <0,0001 |
| Roundness Class II vs. Roundness Class III | -0,5768 | -0,6303 to -0,5233 | Yes | **** | <0,0001 |
| Roundness Class II vs. Roundness Class IV | -0,697 | -0,7520 to -0,6421 | Yes | **** | <0,0001 |
| Roundness Class III vs. Roundness Class IV | -0,1202 | -0,1748 to -0,06571 | Yes | **** | <0,0001 |
| Form-Factor Class I vs. Form-Factor Class II | 0,05647 | 0,01955 to 0,09340 | Yes | *** | 0,0007 |
| Form-Factor Class I vs. Form-Factor Class III | -0,04086 | -0,07754 to -0,004188 | Yes | * | 0,0227 |
| Form-Factor Class I vs. Form-Factor Class IV | 0,006838 | -0,03064 to 0,04432 | No | ns | 0,9641 |
| Form-Factor Class II vs. Form-Factor Class III | -0,09734 | -0,1304 to -0,06425 | Yes | **** | <0,0001 |
| Form-Factor Class II vs. Form-Factor Class IV | -0,04963 | -0,08361 to -0,01566 | Yes | ** | 0,0013 |
| Form-Factor Class III vs. Form-Factor Class IV | 0,0477 | 0,01400 to 0,08140 | Yes | ** | 0,002 |
| Ellipticity Class I vs. Ellipticity Class II | -0,1411 | -0,2123 to -0,06982 | Yes | **** | <0,0001 |
| Ellipticity Class I vs. Ellipticity Class III | -1,055 | -1,126 to -0,9840 | Yes | **** | <0,0001 |
| Ellipticity Class I vs. Ellipticity Class IV | -1,284 | -1,356 to -1,211 | Yes | **** | <0,0001 |
| Ellipticity Class II vs. Ellipticity Class III | -0,9137 | -0,9775 to -0,8498 | Yes | **** | <0,0001 |
| Ellipticity Class II vs. Ellipticity Class IV | -1,143 | -1,208 to -1,077 | Yes | **** | <0,0001 |
| Ellipticity Class III vs. Ellipticity Class IV | -0,229 | -0,2940 to -0,1639 | Yes | **** | <0,0001 |
| P^2^/A Class I vs. P^2^/A Class II | -1,632 | -2,649 to -0,6146 | Yes | *** | 0,0003 |
| P^2^/A Class I vs. P^2^/A Class III | 1,005 | -0,005060 to 2,015 | No | ns | 0,0517 |
| P^2^/A Class I vs. P^2^/A Class IV | -0,1366 | -1,169 to 0,8959 | No | ns | 0,9857 |
| P^2^/A Class II vs. P^2^/A Class III | 2,637 | 1,726 to 3,548 | Yes | **** | <0,0001 |
| P^2^/A Class II vs. P^2^/A Class IV | 1,495 | 0,5594 to 2,431 | Yes | *** | 0,0004 |
| P^2^/A Class III vs. P^2^/A Class IV | -1,142 | -2,070 to -0,2134 | Yes | ** | 0,0094 |
| A/(OLxOH) Class I vs. A/(OLxOH) Class II | -0,01484 | -0,02855 to -0,001121 | Yes | * | 0,0286 |
| A/(OLxOH) Class I vs. A/(OLxOH) Class III | 0,01133 | -0,002289 to 0,02496 | No | ns | 0,1378 |
| A/(OLxOH) Class I vs. A/(OLxOH) Class IV | -0,001515 | -0,01544 to 0,01241 | No | ns | 0,992 |
| A/(OLxOH) Class II vs. A/(OLxOH) Class III | 0,02617 | 0,01388 to 0,03846 | Yes | **** | <0,0001 |
| A/(OLxOH) Class II vs. A/(OLxOH) Class IV | 0,01332 | 0,0007022 to 0,02594 | Yes | * | 0,0343 |
| A/(OLxOH) Class III vs. A/(OLxOH) Class IV | -0,01285 | -0,02537 to -0,0003302 | Yes | * | 0,0419 |
| OW/OL % Class I vs. OW/OL % Class II | -0,0833 | -0,1244 to -0,04219 | Yes | **** | <0,0001 |
| OW/OL % Class I vs. OW/OL % Class III | 0,5049 | 0,4641 to 0,5457 | Yes | **** | <0,0001 |
| OW/OL % Class I vs. OW/OL % Class IV | 0,5543 | 0,5126 to 0,5961 | Yes | **** | <0,0001 |
| OW/OL % Class II vs. OW/OL % Class III | 0,5882 | 0,5514 to 0,6250 | Yes | **** | <0,0001 |
| OW/OL % Class II vs. OW/OL % Class IV | 0,6376 | 0,5998 to 0,6755 | Yes | **** | <0,0001 |
| OW/OL % Class III vs. OW/OL %Class IV | 0,04946 | 0,01194 to 0,08698 | Yes | ** | 0,0046 |
| OL/TL Class I vs. OL/TL Class II | -8,497 | -10,03 to -6,962 | Yes | **** | <0,0001 |
| OL/TL Class I vs. OL/TL Class III | -19,28 | -20,80 to -17,75 | Yes | **** | <0,0001 |
| OL/TL Class I vs. OL/TL Class IV | -27,96 | -29,52 to -26,40 | Yes | **** | <0,0001 |
| OL/TL Class II vs. OL/TL Class III | -10,78 | -12,15 to -9,403 | Yes | **** | <0,0001 |
| OL/TL Class II vs. OL/TL Class IV | -19,46 | -20,88 to -18,05 | Yes | **** | <0,0001 |
| OL/TL Class III vs. OL/TL Class IV | -8,683 | -10,08 to -7,281 | Yes | **** | <0,0001 |
