# Supplementary material for: Intraspecific variability of the saccular and utricular otoliths of the hatchetfish Argyropelecus hemigymnus (Cocco, 1829) from the Strait of Messina (Central Mediterranean Sea)
Source: PLoS One. 2023 Feb 14;18(2):e0281621. doi: 10.1371/journal.pone.0281621 (PMC9928127; doi:10.1371/journal.pone.0281621)
Supplement: S2 Table — (DOCX) [file pone.0281621.s002.docx]

|  | BW vs. otolith.area | BW vs. otolith.length | BW vs. otolith.width | BW vs. otolith.perimeter | BW vs. Roundness | BW vs. Form-Factor | BW vs. Ellipticity | BW vs. P^2^/A | BW vs. A/(OLxOH) | BW vs. OW/OL % | BW vs. OL/TL |
| --- | --- | --- | --- | --- | --- | --- | --- | --- | --- | --- | --- |
| Pearson r |  |  |  |  |  |  |  |  |  |  |  |
| r | 0.902 | 0.9121 | 0.6236 | 0.8707 | 0.8731 | 0.2109 | 0.9008 | -0.2306 | -0.1682 | -0.8507 | 0.9527 |
| 95% confidence interval | 0.8590 to 0.9323 | 0.8733 to 0.9394 | 0.4913 to 0.7278 | 0.8153 to 0.9102 | 0.8187 to 0.9120 | 0.02103 to 0.3862 | 0.8574 to 0.9315 | -0.4036 to -0.04167 | -0.3478 to 0.02334 | -0.8961 to -0.7878 | 0.9312 to 0.9676 |
| R squared | 0.8135 | 0.8319 | 0.3889 | 0.7581 | 0.7623 | 0.0445 | 0.8115 | 0.05317 | 0.02828 | 0.7237 | 0.9077 |
|  |  |  |  |  |  |  |  |  |  |  |  |
| P value |  |  |  |  |  |  |  |  |  |  |  |
| P (two-tailed) | <0.0001 | <0.0001 | <0.0001 | <0.0001 | <0.0001 | 0.03 | <0.0001 | 0.0174 | 0.0849 | <0.0001 | <0.0001 |
| P value summary | **** | **** | **** | **** | **** | * | **** | * | ns | **** | **** |
| Significant? (alpha = 0.05) | Yes | Yes | Yes | Yes | Yes | Yes | Yes | Yes | No | Yes | Yes |
|  |  |  |  |  |  |  |  |  |  |  |  |
| Number of XY Pairs | 106 | 106 | 106 | 106 | 106 | 106 | 106 | 106 | 106 | 106 | 106 |
