# Supplementary material for: Intraspecific variability of the saccular and utricular otoliths of the hatchetfish Argyropelecus hemigymnus (Cocco, 1829) from the Strait of Messina (Central Mediterranean Sea)
Source: PLoS One. 2023 Feb 14;18(2):e0281621. doi: 10.1371/journal.pone.0281621 (PMC9928127; doi:10.1371/journal.pone.0281621)
Supplement: S3 Table — (DOCX) [file pone.0281621.s003.docx]

| < | TL vs. otolith.area | TL vs. otolith.length | TL vs. otolith.width | TL vs. otolith.perimeter | TL vs. Roundness | TL vs. Form-Factor | TL vs. Ellipticity | TL vs. P^2^/A | TL vs. A/(OLxOH) | TL vs. OW/OL % | TL vs. OL/TL |
| --- | --- | --- | --- | --- | --- | --- | --- | --- | --- | --- | --- |
| Pearson r |  |  |  |  |  |  |  |  |  |  |  |
| r | 0.9327 | 0.942 | 0.7163 | 0.9213 | 0.8867 | 0.2125 | 0.9302 | -0.2236 | -0.1569 | -0.8597 | 1 |
| 95% confidence interval | 0.9025 to 0.9537 | 0.9157 to 0.9602 | 0.6087 to 0.7980 | 0.8863 to 0.9458 | 0.8377 to 0.9216 | 0.02265 to 0.3875 | 0.8990 to 0.9521 | -0.3974 to -0.03430 | -0.3375 to 0.03494 | -0.9025 to -0.8002 | 1.000 to 1.000 |
| R squared | 0.8699 | 0.8873 | 0.513 | 0.8488 | 0.7863 | 0.04515 | 0.8654 | 0.04999 | 0.02461 | 0.7391 | 1 |
|  |  |  |  |  |  |  |  |  |  |  |  |
| P value |  |  |  |  |  |  |  |  |  |  |  |
| P (two-tailed) | <0.0001 | <0.0001 | <0.0001 | <0.0001 | <0.0001 | 0.0288 | <0.0001 | 0.0212 | 0.1083 | <0.0001 | <0.0001 |
| P value summary | **** | **** | **** | **** | **** | * | **** | * | ns | **** | **** |
| Significant? (alpha = 0.05) | Yes | Yes | Yes | Yes | Yes | Yes | Yes | Yes | No | Yes | Yes |
|  |  |  |  |  |  |  |  |  |  |  |  |
| Number of XY Pairs | 106 | 106 | 106 | 106 | 106 | 106 | 106 | 106 | 106 | 106 | 106 |
