# Supplementary material for: Intraspecific variability of the saccular and utricular otoliths of the hatchetfish Argyropelecus hemigymnus (Cocco, 1829) from the Strait of Messina (Central Mediterranean Sea)
Source: PLoS One. 2023 Feb 14;18(2):e0281621. doi: 10.1371/journal.pone.0281621 (PMC9928127; doi:10.1371/journal.pone.0281621)
Supplement: S4 Table — (DOCX) [file pone.0281621.s004.docx]

|  |  |  |  |  |
| --- | --- | --- | --- | --- |
| **Classes** | **Comparisons** | **P Value** | **Df** | **F** |
| I | left *vs* right | 0.001 | 1 | 4.5 |
| II | left *vs* right | 0.18 |  |  |
| III | left *vs* right | 0.013 | 1 | 2.8 |
| IV | left *vs* right | 0.021 | 1 | 2.4 |
