# Supplementary material for: Intraspecific variability of the saccular and utricular otoliths of the hatchetfish Argyropelecus hemigymnus (Cocco, 1829) from the Strait of Messina (Central Mediterranean Sea)
Source: PLoS One. 2023 Feb 14;18(2):e0281621. doi: 10.1371/journal.pone.0281621 (PMC9928127; doi:10.1371/journal.pone.0281621)
Supplement: S5 Table — (DOCX) [file pone.0281621.s005.docx]

|  |  |  |  |  |  |
| --- | --- | --- | --- | --- | --- |
| **Tukey's multiple comparisons test** | **Mean Diff.** | **95,00% CI of diff.** | **Significant?** | **Summary** | **Adjusted P Value** |
| Area Class I vs. Area Class II | -0.01578 | -0.02188 to -0.009686 | Yes | **** | <0.0001 |
| Area Class I vs. Area Class III | -0.0421 | -0.04826 to -0.03594 | Yes | **** | <0.0001 |
| Area Class I vs. Area Class IV | -0.05506 | -0.06249 to -0.04764 | Yes | **** | <0.0001 |
| Area Class II vs. Area Class III | -0.02632 | -0.03180 to -0.02084 | Yes | **** | <0.0001 |
| Area Class II vs. Area Class IV | -0.03928 | -0.04615 to -0.03241 | Yes | **** | <0.0001 |
| Area Class III vs. Area Class IV | -0.01296 | -0.01989 to -0.006035 | Yes | **** | <0.0001 |
| Length Class I vs. Length Class II | -0.04546 | -0.06384 to -0.02707 | Yes | **** | <0.0001 |
| Length Class I vs. Length Class III | -0.1065 | -0.1251 to -0.08794 | Yes | **** | <0.0001 |
| Length Class I vs. Length Class IV | -0.1472 | -0.1695 to -0.1248 | Yes | **** | <0.0001 |
| Length Class II vs. Length Class III | -0.06105 | -0.07758 to -0.04452 | Yes | **** | <0.0001 |
| Length Class II vs. Length Class IV | -0.1017 | -0.1224 to -0.08099 | Yes | **** | <0.0001 |
| Length Class III vs. Length Class IV | -0.04065 | -0.06153 to -0.01977 | Yes | **** | <0.0001 |
| Width Class I vs. Width Class II | -0.05316 | -0.06969 to -0.03662 | Yes | **** | <0.0001 |
| Width Class I vs. Width Class III | -0.1231 | -0.1398 to -0.1064 | Yes | **** | <0.0001 |
| Width Class I vs. Width Class IV | -0.1517 | -0.1718 to -0.1316 | Yes | **** | <0.0001 |
| Width Class II vs. Width Class III | -0.06992 | -0.08479 to -0.05506 | Yes | **** | <0.0001 |
| Width Class II vs. Width Class IV | -0.09855 | -0.1172 to -0.07992 | Yes | **** | <0.0001 |
| Width Class III vs. Width Class IV | -0.02862 | -0.04740 to -0.009844 | Yes | *** | 0.0009 |
| Perimeter Class I vs. Perimeter Class II | -0.1611 | -0.2114 to -0.1107 | Yes | **** | <0.0001 |
| Perimeter Class I vs. Perimeter Class III | -0.3816 | -0.4325 to -0.3307 | Yes | **** | <0.0001 |
| Perimeter Class I vs. Perimeter Class IV | -0.4819 | -0.5432 to -0.4205 | Yes | **** | <0.0001 |
| Perimeter Class II vs. Perimeter Class III | -0.2205 | -0.2658 to -0.1752 | Yes | **** | <0.0001 |
| Perimeter Class II vs. Perimeter Class IV | -0.3208 | -0.3775 to -0.2640 | Yes | **** | <0.0001 |
| Perimeter Class III vs. Perimeter Class IV | -0.1003 | -0.1575 to -0.04303 | Yes | *** | 0.0001 |
| Roundness Class I vs. Roundness Class II | 0.04036 | -0.05548 to 0.1362 | No | ns | 0.6822 |
| Roundness Class I vs. Roundness Class III | 0.08401 | -0.01282 to 0.1808 | No | ns | 0.111 |
| Roundness Class I vs. Roundness Class IV | 0.08336 | -0.03329 to 0.2000 | No | ns | 0.2433 |
| Roundness Class II vs. Roundness Class III | 0.04365 | -0.04254 to 0.1298 | No | ns | 0.5415 |
| Roundness Class II vs. Roundness Class IV | 0.043 | -0.06498 to 0.1510 | No | ns | 0.7186 |
| Roundness Class III vs. Roundness Class IV | -0.0006457 | -0.1095 to 0.1082 | No | ns | >0.9999 |
| Form-Factor Class I vs. Form-Factor Class II | 0.001079 | -0.01466 to 0.01682 | No | ns | 0.9979 |
| Form-Factor Class I vs. Form-Factor Class III | 0.02745 | 0.01155 to 0.04335 | Yes | *** | 0.0002 |
| Form-Factor Class I vs. Form-Factor Class IV | 0.0466 | 0.02744 to 0.06576 | Yes | **** | <0.0001 |
| Form-Factor Class II vs. Form-Factor Class III | 0.02637 | 0.01221 to 0.04053 | Yes | **** | <0.0001 |
| Form-Factor Class II vs. Form-Factor Class IV | 0.04552 | 0.02779 to 0.06326 | Yes | **** | <0.0001 |
| Form-Factor Class III vs. Form-Factor Class IV | 0.01915 | 0.001275 to 0.03703 | Yes | * | 0.0313 |
| Ellipticity Class I vs. Ellipticity Class II | -0.05956 | -0.1507 to 0.03163 | No | ns | 0.3187 |
| Ellipticity Class I vs. Ellipticity Class III | -0.1555 | -0.2477 to -0.06341 | Yes | *** | 0.0002 |
| Ellipticity Class I vs. Ellipticity Class IV | -0.2627 | -0.3737 to -0.1517 | Yes | **** | <0.0001 |
| Ellipticity Class II vs. Ellipticity Class III | -0.09598 | -0.1780 to -0.01398 | Yes | * | 0.0156 |
| Ellipticity Class II vs. Ellipticity Class IV | -0.2032 | -0.3059 to -0.1004 | Yes | **** | <0.0001 |
| Ellipticity Class III vs. Ellipticity Class IV | -0.1072 | -0.2108 to -0.003628 | Yes | * | 0.0398 |
| P^2^/A Class I vs. P^2^/A Class II | -0.01362 | -0.2392 to 0.2119 | No | ns | 0.9985 |
| P^2^/A Class I vs. P^2^/A Class III | -0.3851 | -0.6130 to -0.1573 | Yes | *** | 0.0002 |
| P^2^/A Class I vs. P^2^/A Class IV | -0.6591 | -0.9336 to -0.3845 | Yes | **** | <0.0001 |
| P^2^/A Class II vs. P^2^/A Class III | -0.3715 | -0.5744 to -0.1687 | Yes | **** | <0.0001 |
| P^2^/A Class II vs. P^2^/A Class IV | -0.6454 | -0.8996 to -0.3913 | Yes | **** | <0.0001 |
| P^2^/A Class III vs. P^2^/A Class IV | -0.2739 | -0.5301 to -0.01772 | Yes | * | 0.0317 |
| A/(OLxOH) Class I vs. A/(OLxOH) Class II | -0.006331 | -0.02871 to 0.01605 | No | ns | 0.8768 |
| A/(OLxOH) Class I vs. A/(OLxOH) Class III | -0.001945 | -0.02456 to 0.02067 | No | ns | 0.9958 |
| A/(OLxOH) Class I vs. A/(OLxOH) Class IV | 0.02972 | 0.002476 to 0.05696 | Yes | * | 0.0273 |
| A/(OLxOH) Class II vs. A/(OLxOH) Class III | 0.004386 | -0.01574 to 0.02451 | No | ns | 0.9386 |
| A/(OLxOH) Class II vs. A/(OLxOH) Class IV | 0.03605 | 0.01083 to 0.06126 | Yes | ** | 0.0021 |
| A/(OLxOH) Class III vs. A/(OLxOH) Class IV | 0.03166 | 0.006241 to 0.05708 | Yes | ** | 0.0089 |
| OW/OL % Class I vs. OW/OL % Class II | -0.03906 | -0.1238 to 0.04564 | No | ns | 0.6166 |
| OW/OL % Class I vs. OW/OL % Class III | -0.07406 | -0.1596 to 0.01151 | No | ns | 0.1123 |
| OW/OL % Class I vs. OW/OL % Class IV | -0.03613 | -0.1392 to 0.06696 | No | ns | 0.7902 |
| OW/OL % Class II vs. OW/OL % Class III | -0.035 | -0.1112 to 0.04116 | No | ns | 0.6193 |
| OW/OL % Class II vs. OW/OL % Class IV | 0.002928 | -0.09250 to 0.09835 | No | ns | 0.9998 |
| OW/OL % Class III vs. OW/OL %Class IV | 0.03793 | -0.05827 to 0.1341 | No | ns | 0.7248 |
| OL/TL Class I vs. OL/TL Class II | -8.436 | -10.37 to -6.502 | Yes | **** | <0.0001 |
| OL/TL Class I vs. OL/TL Class III | -19.77 | -21.72 to -17.81 | Yes | **** | <0.0001 |
| OL/TL Class I vs. OL/TL Class IV | -28.04 | -30.39 to -25.68 | Yes | **** | <0.0001 |
| OL/TL Class II vs. OL/TL Class III | -11.33 | -13.07 to -9.590 | Yes | **** | <0.0001 |
| OL/TL Class II vs. OL/TL Class IV | -19.6 | -21.78 to -17.42 | Yes | **** | <0.0001 |
| OL/TL Class III vs. OL/TL Class IV | -8.272 | -10.47 to -6.075 | Yes | **** | <0.0001 |
