# Supplementary material for: Intraspecific variability of the saccular and utricular otoliths of the hatchetfish Argyropelecus hemigymnus (Cocco, 1829) from the Strait of Messina (Central Mediterranean Sea)
Source: PLoS One. 2023 Feb 14;18(2):e0281621. doi: 10.1371/journal.pone.0281621 (PMC9928127; doi:10.1371/journal.pone.0281621)
Supplement: S6 Table — (DOCX) [file pone.0281621.s006.docx]

|  | BW vs. otolith.area | BW vs. otolith.length | BW vs. otolith.width | BW vs. otolith.perimeter | BW vs. Roundness | BW vs. Form-Factor | BW vs. Ellipticity | BW vs. P2/A | BW vs. A/(OLxOH) | BW vs. OW/OL % | BW vs. OL/TL |
| --- | --- | --- | --- | --- | --- | --- | --- | --- | --- | --- | --- |
| Pearson r |  |  |  |  |  |  |  |  |  |  |  |
| r | 0.8856 | 0.8725 | 0.8671 | 0.8757 | -0.2758 | -0.7106 | 0.6464 | 0.7065 | -0.3711 | 0.1517 | 0.955 |
| 95% confidence interval | 0.8157 to 0.9300 | 0.7955 to 0.9218 | 0.7871 to 0.9184 | 0.8003 to 0.9238 | -0.4934 to -0.02581 | -0.8163 to -0.5588 | 0.4713 to 0.7725 | 0.5531 to 0.8136 | -0.5697 to -0.1316 | -0.1041 to 0.3886 | 0.9258 to 0.9728 |
| R squared | 0.7843 | 0.7613 | 0.7518 | 0.7668 | 0.07609 | 0.5049 | 0.4178 | 0.4992 | 0.1377 | 0.023 | 0.912 |
|  |  |  |  |  |  |  |  |  |  |  |  |
| P value |  |  |  |  |  |  |  |  |  |  |  |
| P (two-tailed) | <0.0001 | <0.0001 | <0.0001 | <0.0001 | 0.0314 | <0.0001 | <0.0001 | <0.0001 | 0.0032 | 0.2433 | <0.0001 |
| P value summary | **** | **** | **** | **** | * | **** | **** | **** | ** | ns | **** |
| Significant? (alpha = 0.05) | Yes | Yes | Yes | Yes | Yes | Yes | Yes | Yes | Yes | No | Yes |
|  |  |  |  |  |  |  |  |  |  |  |  |
| Number of XY Pairs | 61 | 61 | 61 | 61 | 61 | 61 | 61 | 61 | 61 | 61 | 61 |
