# Supplementary material for: Intraspecific variability of the saccular and utricular otoliths of the hatchetfish Argyropelecus hemigymnus (Cocco, 1829) from the Strait of Messina (Central Mediterranean Sea)
Source: PLoS One. 2023 Feb 14;18(2):e0281621. doi: 10.1371/journal.pone.0281621 (PMC9928127; doi:10.1371/journal.pone.0281621)
Supplement: S7 Table — (DOCX) [file pone.0281621.s007.docx]

|  | TL vs. otolith.area | TL vs. otolith.length | TL vs. otolith.width | TL vs. otolith.perimeter | TL vs. Roundness | TL vs. Form-Factor | TL vs. Ellipticity | TL vs. P2/A | TL vs. A/(OLxOH) | TL vs. OW/OL % | TL vs. OL/TL |
| --- | --- | --- | --- | --- | --- | --- | --- | --- | --- | --- | --- |
| Pearson r |  |  |  |  |  |  |  |  |  |  |  |
| r | 0.9175 | 0.9021 | 0.9244 | 0.9196 | -0.3387 | -0.6688 | 0.6294 | 0.665 | -0.3327 | 0.2289 | 1 |
| 95% confidence interval | 0.8656 to 0.9498 | 0.8414 to 0.9403 | 0.8766 to 0.9541 | 0.8690 to 0.9512 | -0.5441 to -0.09494 | -0.7879 to -0.5014 | 0.4486 to 0.7606 | 0.4963 to 0.7853 | -0.5393 to -0.08822 | -0.02436 to 0.4545 | 1.000 to 1.000 |
| R squared | 0.8417 | 0.8137 | 0.8545 | 0.8457 | 0.1147 | 0.4472 | 0.3961 | 0.4422 | 0.1107 | 0.05238 | 1 |
|  |  |  |  |  |  |  |  |  |  |  |  |
| P value |  |  |  |  |  |  |  |  |  |  |  |
| P (two-tailed) | <0.0001 | <0.0001 | <0.0001 | <0.0001 | 0.0076 | <0.0001 | <0.0001 | <0.0001 | 0.0088 | 0.076 | <0.0001 |
| P value summary | **** | **** | **** | **** | ** | **** | **** | **** | ** | ns | **** |
| Significant? (alpha = 0.05) | Yes | Yes | Yes | Yes | Yes | Yes | Yes | Yes | Yes | No | Yes |
|  |  |  |  |  |  |  |  |  |  |  |  |
| Number of XY Pairs | 61 | 61 | 61 | 61 | 61 | 61 | 61 | 61 | 61 | 61 | 61 |
