# Supplementary figures and images for: Intraspecific variability of the saccular and utricular otoliths of the hatchetfish Argyropelecus hemigymnus (Cocco, 1829) from the Strait of Messina (Central Mediterranean Sea)
Source: PLoS One. 2023 Feb 14;18(2):e0281621. doi: 10.1371/journal.pone.0281621 (PMC9928127; doi:10.1371/journal.pone.0281621)

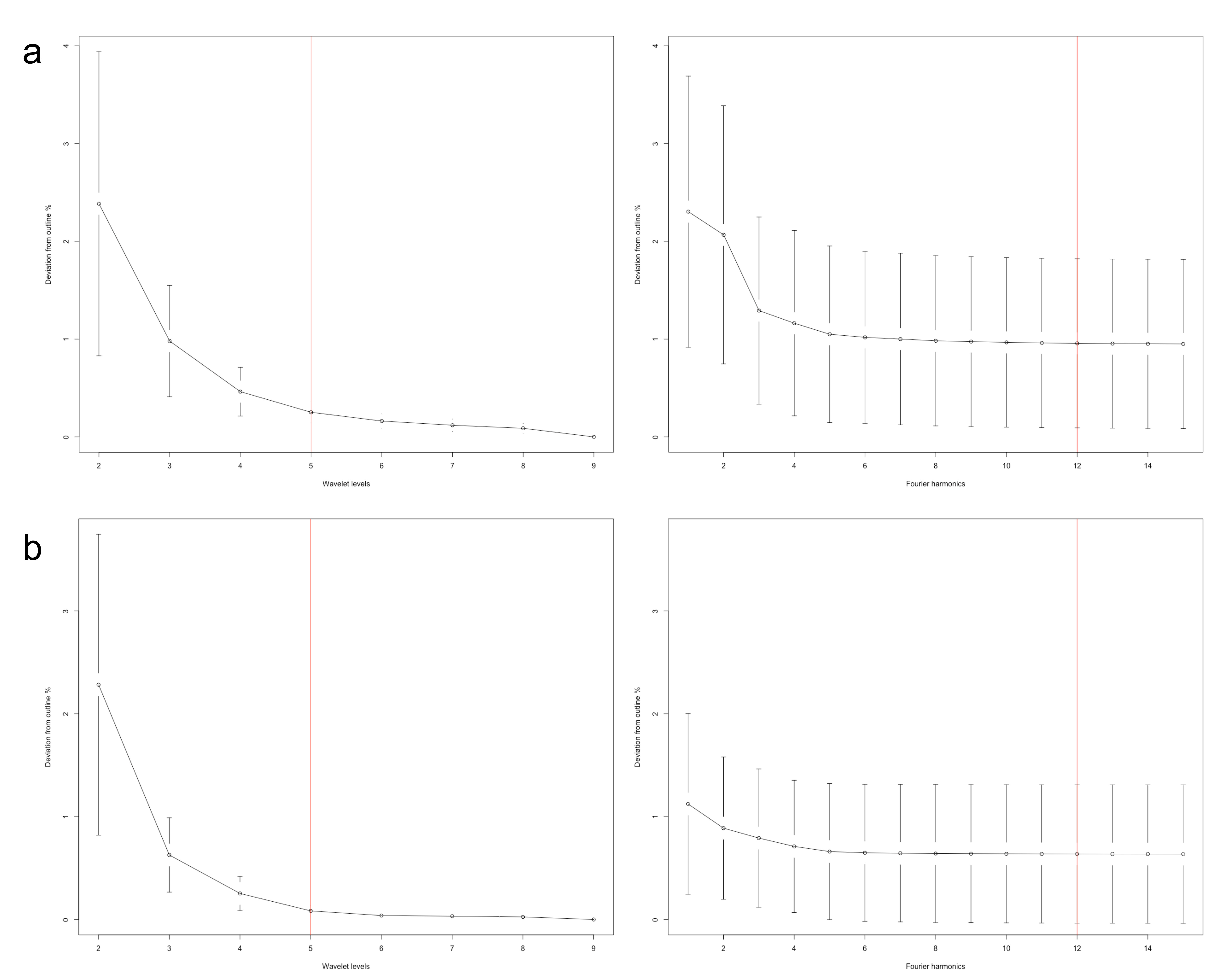

Supplement: S1 Fig — The red lines indicate the level of Wavelet and number of Fourier harmonics needed for a 98.5% accuracy of the remodelling. (TIF) [file pone.0281621.s008.tif]

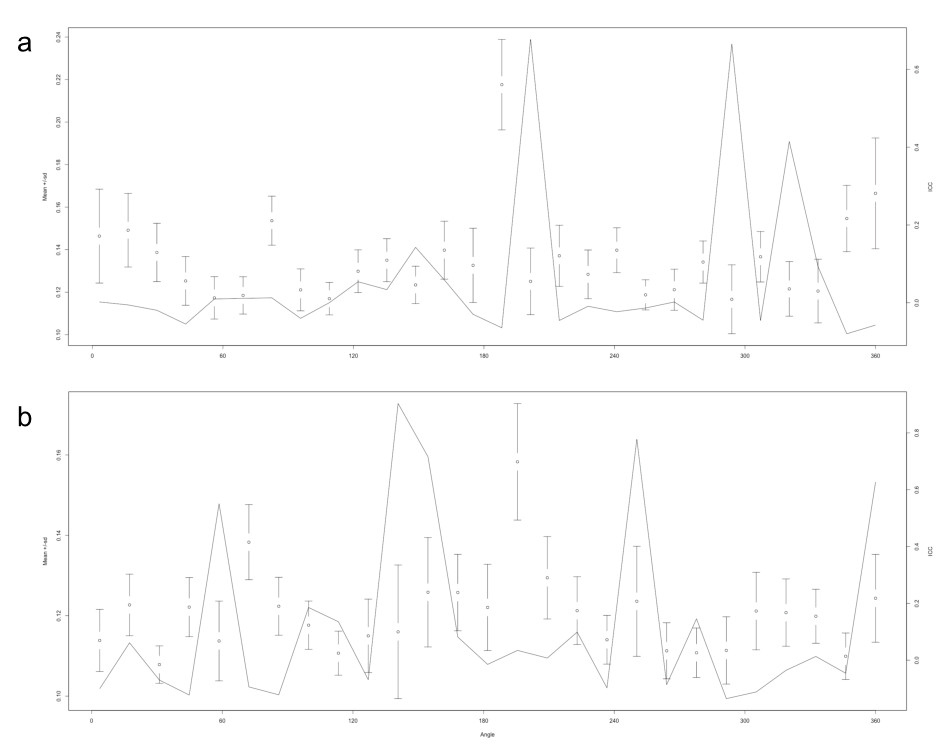

Supplement: S2 Fig — The horizontal axis shows angle in degrees (°) based on the polar coordinates of the mean otoliths shape plot. The centroid of the otolith is the center point of polar coordinates. (TIF) [file pone.0281621.s009.tif]
